# Imagining is Not Doing but Involves Specific Motor Commands: A Review of Experimental Data Related to Motor Inhibition

**DOI:** 10.3389/fnhum.2012.00247

**Published:** 2012-09-05

**Authors:** Aymeric Guillot, Franck Di Rienzo, Tadhg MacIntyre, Aidan Moran, Christian Collet

**Affiliations:** ^1^Centre de Recherche et d’Innovation sur le Sport (EA 647), équipe Performance Motrice, Mentale et du Matériel, Université de Lyon, Université Claude Bernard Lyon 1Villeurbanne, France; ^2^Institut Universitaire de FranceParis, France; ^3^Physical Education and Sport Sciences Department, University of LimerickLimerick, Ireland; ^4^School of Psychology, University College DublinDublin, Ireland

**Keywords:** motor imagery, motor command inhibition, motor performance, mental processes, electromyography, sensorimotor control

## Abstract

There is now compelling evidence that motor imagery (MI) and actual movement share common neural substrate. However, the question of how MI inhibits the transmission of motor commands into the efferent pathways in order to prevent any movement is largely unresolved. Similarly, little is known about the nature of the electromyographic activity that is apparent during MI. In addressing these gaps in the literature, the present paper argues that MI includes motor execution commands for muscle contractions which are blocked at some level of the motor system by inhibitory mechanisms. We first assemble data from neuroimaging studies that demonstrate that the neural networks mediating MI and motor performance are not totally overlapping, thereby highlighting potential differences between MI and actual motor execution. We then review MI data indicating the presence of subliminal muscular activity reflecting the intrinsic characteristics of the motor command as well as increased corticomotor excitability. The third section not only considers the inhibitory mechanisms involved during MI but also examines how the brain resolves the problem of issuing the motor command for action while supervising motor inhibition when people engage in voluntary movement during MI. The last part of the paper draws on imagery research in clinical contexts to suggest that some patients move while imagining an action, although they are not aware of such movements. In particular, experimental data from amputees as well as from patients with Parkinson’s disease are discussed. We also review recent studies based on comparing brain activity in tetraplegic patients with that from healthy matched controls that provide insights into inhibitory processes during MI. We conclude by arguing that based on available evidence, a multifactorial explanation of motor inhibition during MI is warranted.

## Introduction

One of the most remarkable capacities of the mind is its ability to simulate sensations, movements, and other types of experience. In most occasions, mentally imagining is like perceiving in the absence of the corresponding sensory information. In other words, imagining involves “seeing” with the “mind’s eye,” “hearing” with the “mind’s ear,” and so on for each sensory modality (Kosslyn, 2010). Accordingly, mental imagery is a multimodal construct which consists of either recalling previously perceived images or feelings, or envisaging forthcoming events. Within this construct, “motor imagery” (MI) refers to the mental representation of an action without engaging in its actual execution. MI involves an integrated covert simulation of physical movement, and may be defined as a dynamic mental state during which the representation of a given motor act is rehearsed in working memory without any overt motor output (Decety and Grezes, 1999; Collet and Guillot, 2010).

The vast majority of experimental investigations dealing with the MI experience primarily focused on visual and kinesthetic imagery (KI). While visual imagery (VI) refers as to the visualization of an action, KI involves the sensations of how it feels to perform an action, including the force and effort perceived during movement and balance (Callow and Waters, 2005), hence suggesting to consider the body as a generator of forces (Jeannerod, 1994). Interestingly, previous data showed that KI was close to motor execution, with an extensive overlap of the corresponding neural networks (Solodkin et al., 2004). Other researchers introduced the concept of imagery perspective and further distinguished between first- and third-person VI perspectives. During the first-person perspective, performers visualize the action as how would happen in the real-life situation, while in the third-person perspective, they imagine, as spectators, the action that somebody is performing, regardless of the agency of the movement (i.e., whether they, “see” themselves or others). Although there is some confusion in the usage of these terms in many studies (Moran et al., 2012), a considerable amount of experimental research suggests that MI is a valuable and cost-effective technique to improve motor performance and to enhance motor recovery (see reviews by Driskell et al., 1994; de Vries and Mulder, 2007; Guillot and Collet, 2008; Munzert et al., 2009).

Despite the preceding evidence on the efficacy of MI, several unresolved issues have emerged with regard to the neural underpinnings of this construct. More precisely, little agreement exists among imagery researchers as to the extent to which the neural substrates of MI overlap with those of actual practice. Also, little evidence exists on the question of whether or not motor commands are inhibited during imagined movements (Kasess et al., 2008). Nevertheless, certain trends exist in the relevant literature. For example, the pattern of electromyographic (EMG) activity during MI generally supports the hypothesis of residual muscle activity which might originate from an incomplete inhibition of the motor command (Jeannerod, 1994). Similarly, transcranial magnetic stimulation (TMS) experiments also support this assumption by delineating the features of corticospinal facilitation during MI (Stinear, 2010). Finally, clinical studies show that some patients with specific brain damage fail to inhibit the motor action associated with its mental representation, and thus fully “execute the imagined action,” hence highlighting uninhibited movements during mental rehearsal. Data in amputees, patients with spinal cord injury (SCI) and Parkinson’s disease (PD) further contribute to theoretical understanding of how the motor command is inhibited during MI.

Against this background, the present paper aims to examine these issues and provide deeper evidence that MI includes motor commands for muscle contractions, which are blocked at some level of the motor system by inhibitory mechanisms. On some occasions (e.g., in sport settings), individuals may retain the potential to move while imagining an action, e.g., miming unambiguously some parts of movement execution while rehearsing the imagined movement. These situations raise an important but neglected question: How does the brain resolve the paradox whereby it is required to issue the motor command needed for action when MI is performed, while concurrently issuing an inhibitory command when the person is moving during MI?

## Neural Correlates of Motor Imagery

Understanding the neural correlates of motor performance and MI has been an important purpose of brain studies since the advent of neuroimaging techniques. Considerable experimental evidence has accumulated to suggest that movement execution and MI share substantial overlap of active brain regions (for review, see Guillot et al., 2012). Such apparent functional equivalence supports the hypothesis that MI draws on the similar neural networks that are used in actual perception and motor control (Jeannerod, 1994; Grezes and Decety, 2001; Holmes and Collins, 2001). Moreover, MI can also activate neural circuits used during tasks investigating memory and emotion (Kosslyn et al., 2001). As we will see, however, the neural networks underlying these behavior are not strictly identical. This is because when performing MI, participants are aware that movement will not be performed, and therefore that motor commands must be inhibited.

Neuroimaging studies provided preliminary evidence that motor-related areas of the brain (e.g., the ventral and dorsal parts of the premotor cortex, as well as the supplementary motor area – SMA) and subcortical areas including the cerebellum and the basal ganglia, are active during MI of both simple and complex movements (e.g., Lotze and Halsband, 2006; Guillot et al., 2008; Munzert et al., 2009). Furthermore, research suggests that MI activates a subset of areas required for movement execution (Macuga and Frey, 2012), thus leading to a partial overlap in the corresponding neural networks. The contribution of the contralateral primary motor cortex (cM1) to imagined actions is more controversial, however (for reviews, see Lotze and Halsband, 2006; Munzert et al., 2009; Lotze and Zentgraf, 2010). Whereas some researchers did not report cM1 activations during MI (e.g., Gerardin et al., 2000; Hanakawa et al., 2008), others found fleeting involvement (Dechent et al., 2004) or significant activation (Lotze et al., 1999b; Porro et al., 2000; Solodkin et al., 2004; Guillot et al., 2008; Sharma et al., 2008). Such discrepancies may be due to methodological differences and difficulties in monitoring compliance with MI instructions (Sharma et al., 2006). Interestingly, Ehrsson et al. (2003) showed that the content of MI was reflected in the pattern of motor cortical activation, as MI of hand, foot, and tongue movements specifically activated the corresponding hand, foot, and tongue sections of cM1. Additional evidence indicates that activation of cM1 might be differentially influenced by MI instructions, MI ability, and motor expertise (Lotze and Zentgraf, 2010). Taken together, the bulk of neuroimaging studies suggest that cM1 is activated during MI – but more weakly than during actual movement. Interestingly, Kasess et al. (2008) reported that SMA may substantially contribute to inhibit activity of cM1 during MI.

Activation of parietal areas including the inferior and superior parietal lobules, as well as the precuneus, was also frequently reported during MI (Gerardin et al., 2000; Hanakawa et al., 2003; Guillot et al., 2009; Munzert et al., 2009). Experimental studies in patients with parietal lesions further support that these structures are critically involved in the generation and guidance of mental images, including the ability to achieve temporal congruence between MI and motor performance (Sirigu et al., 1996; Malouin et al., 2004).

The patterns of neural activity underlying imagery types (e.g., VI vs. KI) and imagery perspectives (first-person vs. third-person imagery) are partially mediated through separate neural systems. For instance, Solodkin et al. (2004) investigated neural networks associated with physical execution, VI, and KI of hand movements. Although some shared neural substrates were evident between these processes, differences were found in the inputs received from the superior parietal lobule. Specifically, inputs from SMA to cM1 were lower to those observed during motor execution (Gao et al., 2011). In a single group of participants with high MI abilities, Guillot et al. (2009) showed that VI activated predominantly the visual pathways including the occipital regions and the precuneus, whereas KI involved mainly motor-associated structures and the inferior parietal lobule.

Neuroimaging studies have also shown that the neural networks underlying MI differ as a function of both individual expertise level and imagery ability. For instance, an inverse relationship between the pattern of brain activity and expertise level has been reported (Ross et al., 2003; Milton et al., 2007) with decreased activations in the SMA, the cerebellum, and the basal ganglia. More recent work by Guillot et al. (2008) confirms that the neural networks mediating MI partially differ as a function of imagery ability. Specifically, whereas strong imagers tended to show activation in the parietal and ventrolateral premotor regions, weaker imagers tended to recruit the cerebellum, the orbito-frontal, and posterior cingulate cortices. In both cases, however, dynamic brain changes were found to become more refined and circumscribed with imagery/physical practice (PP) – a trend which was also evident during the learning process of motor tasks.

In summary, neuroimaging research clearly demonstrates that MI and motor performance of the same task share certain neural substrates but the overlap is incomplete. Furthermore, data challenge the assumption that neural activity is described as being in all of the same areas as execution, albeit to a lesser extent. As expected, some areas that are active during motor performance are not involved during MI. Although less common, the converse is also true, with some regions being more strongly and/or selectively activated during MI compared to actual execution of the same movement (e.g., the pre-SMA – e.g., Hanakawa et al., 2008). From the preceding evidence, we conclude that the main difference between MI and motor performance is probably that MI involves the *inhibition* of some motor commands triggering movements – although the neural *level* at which the motor command is stopped has not yet been clearly identified. To address this latter issue, we believe that EMG recordings may provide a reliable means to investigate whether or not the brain activation recorded during MI actually originate from mental representation – as opposed to the potential motor activity that could have accompanied the task. In a similar vein, TMS can be used to explore the degree to which MI modulates both corticomotor excitability and intracortical inhibition.

## Muscle and TMS Activity during Imagined Actions

### EMG correlates of motor imagery

A great amount of experimental data has been collected on the physiological operations involved during MI (Table 1) – notably the peripheral muscular activity which may occur during the mental representation of an action (Guillot et al., 2010). Since the pioneering work of Jacobson (1930, 1932), who provided the first scientific evidence that MI of bending the arm produced small contractions of the flexor arm muscles, debate has existed on whether or not MI is accompanied by subliminal muscle contractions.

**Table 1:** **Studies investigating the EMG activity during motor imagery**.

Study	Number of participants	Motor task	Main findings
**LACK OF MUSCLE ACTIVITY DURING MOTOR IMAGERY**			
Decety et al. (1993)	*n* = 6	Leg contraction to press and release a loaded footplate	No change in phosphocreatine concentration or in pH during motor imagery
Demougeot and Papaxanthis (2011)	*n* = 17	Vertical arm movements	No arm muscle activation during motor imagery
Gentili et al. (2006)	*n* = 40	Pointing arm movement	No EMG activity during motor imagery
Gerardin et al. (2000)	*n* = 8	Auditory-cued hand movements	Surface EMG did not detect any muscle activity during motor imagery
Gueugneau et al. (2008)	*n* = 9	Pointing arm movement	No EMG activity during motor imagery
Hanakawa et al. (2008)	*n* = 13	Finger tapping sequence	Surface EMG was monitored during fMRI recordings to confirm the absence of muscle activity during motor imagery
Jackson et al. (2003)	*n* = 9	Foot sequence task	No significant difference in the EMG signal between imagery and baseline conditions, showing that the patterns of cerebral activation during fMRI recordings are not due to movements
Kleber et al. (2007)	*n* = 16	Singing of an Italian aria	No difference between baseline and imagined singing
Lafleur et al. (2002)	*n* = 9	Foot sequence task	EMG recordings showed no change in muscle activity during scans compared to baseline levels
Lim et al. (2006)	*n* = 13	Arm movement	No EMG activity during motor imagery
Lotze et al. (1999b)	*n* = 10	Making a fist	Low EMG activity, which did not differ from the baseline, was a precondition before fMRI recordings
Lotze et al. (2003)	*n* = 16	Performance of Mozart’s violin concerto in G major	No observable differences between motor imagery and rest
Mulder et al. (2004)	*n* = 37	Abduction of the big toe	No EMG activity during motor imagery
Mulder et al. (2005)	*n* = 31	Squat movements with additional weights	EMG activity recorded during motor imagery did not differ from baseline
Naito et al. (2002)	*n* = 10	Palmar flexion and dorsiflexion of the wrist	No EMG activity in the motor imagery condition
Personnier et al. (2008)	*n* = 28	Arm movements in the sagittal plane	Muscle activation patterns are very similar between motor imagery and rest conditions
Ranganathan et al. (2004)	*n* = 30	Isometric little finger abduction and elbow flexion	Muscle activity during motor imagery was near zero
Roosink and Zijdewind (2010)	*n* = 20	Finger tapping sequence	No EMG activity during motor imagery
Shick (1970)	*n* = 10	Volleyball serve skill	No EMG activity during motor imagery
Yahagi et al. (1996)	*n* = 7	Wrist flexion	No EMG activity during motor imagery
Yue and Cole (1992)	*n* = 30	Isometric little finger abduction	No EMG activity during motor imagery
Zijdewind et al. (2003)	*n* = 29	Ankle plantar-flexion	No EMG activity during motor imagery in the majority of the participants. When little EMG activity was recorded, participants were asked to concentrate until being able imagining the movement without muscle activation
**MUSCLE ACTIVITY DURING MOTOR IMAGERY**			
Bird (1984)	*n* = 5	Motor imagery of a past athletic event (including riding, rowing, swimming, water skiing, and basketball)	The EMG configuration during motor imagery mirrored that observed during actual practice
Bonnet et al. (1997)	*n* = 26	Foot pressure on a pedal	EMG activity weakly increased during motor imagery
Boschker (2001) Bakker et al. (1996)	*n* = 39	Arm lifting movements (biceps curls)	Significant EMG activity is recorded in the muscles contributing to the contraction. Greater muscle activity in the active than in the passive arm, and greater biceps activity when imagining lifting a heavy compared to a light weight
Dickstein et al. (2005)	*n* = 15	Rising on tiptoes	EMG activity was recorded in six participants in at least one of the target muscles
Gandevia et al. (1997)	*n* = 12	Range of simple and complex movements (e.g., flexions/extensions, handwriting, walking, threading a needle…)	Imagery increased background EMG in the involved muscles. In some occasions, spindle discharge also increased
Guillot et al. (2007)	*n* = 30	Biceps dumbbell curls	The magnitude of EMG activity is correlated to the mental effort required to imagine the movement. EMG patterns during imagery of concentric, isometric, and eccentric contractions mirror those observed during actual movements. EMG activity is recorded in agonist, antagonist, synergist, and fixator muscles
Hale (1982)	*n* = 48	Biceps dumbbell curls	Internal imagery perspective produced greater biceps activity than the external imagery perspective
Harris and Robinson (1986)	*n* = 36	Arm lifting	Significant EMG activity is recorded in the muscles contributing to the contraction. Greater EMG activity during the first-person than during the third-person perspective
Hashimoto and Rothwell (1999)	*n* = 9	Wrist flexion and extension	Larger EMG responses in flexor and extensor muscles during imagined flexions and extensions, respectively
Jacobson (1930, 1932)	The number of participants varied among tasks	Biceps dumbbell curls, bending the forearm, sweeping, climbing a rope	EMG activity was recorded in the specific muscle involved with the imagined activity
Jowdy and Harris (1990)	*n* = 38	Juggling task	Increased muscle activity during motor imagery. No effect of the imagery ability on the magnitude of muscle activity
Lebon et al. (2008)	*n* = 30	Biceps dumbbell curls	The median frequency of EMG power spectrum in agonist and antagonist muscles was significantly higher during motor imagery than during baseline
Li et al. (2004a)	*n* = 9	Flexion and extension movements of the fingers	EMG activity was recorded in the finger flexors in four participants
Livesay and Samaras (1998)	*n* = 30	Tightly squeezing a hand-size rubber ball	Increased EMG activity in the dominant forearm
Lutz and Linder (2001)	*n* = 160	Dart throwing	Greater biceps EMG activity was recorded when imagery instructions included assertions about behavior, such as motor actions and visceral responses
Shaw (1938)	The number of participants varied among tasks	Range of complex movements (e.g., flexions/extensions, handwriting, walking, threading a needle…)	Increased EMG activity during motor imagery was distributed across different muscle groups including those not directly related to the corresponding movement
Slade et al. (2002)	*n* = 60	Biceps dumbbell and manipulandum curls	EMG activity was significantly greater for both curls in the active arm during motor imagery when compared to baseline
Suinn (1980)	*n* = 1	Skiing a downhill race	Recorded muscle patterns were strikingly similar to those observed during actual practice
Wehner et al. (1984)	*n* = 27	Contour tracking arm task	Similar frequency distribution in the power spectrum during actual practice and motor imagery

Muscle quiescence during MI has been reported in many experimental studies (e.g., Yue and Cole, 1992; Decety et al., 1993; Lotze et al., 1999b; Mulder et al., 2005). Interestingly, the lack of EMG activity during MI was sometimes considered a precondition prior to engaging in MI practice (Michelon et al., 2006). On some occasions, EMG data were even recorded during scanning sessions *per se*, to demonstrate that variations of cerebral blood flow were directly related to the mental work and not to any concomitant movement (e.g., Gerardin et al., 2000; Jackson et al., 2003; Lotze et al., 2003; Hanakawa et al., 2008). In sum, these results seem to suggest that MI involves the intention of *not* executing the movement, with strong inhibitory mechanisms blocking the motor command.

Conversely, similar EMG activity has been observed during overt motor execution and MI conditions, with a reduced magnitude in the simulated action (e.g., Jowdy and Harris, 1990; Hashimoto and Rothwell, 1999). Gandevia et al. (1997) further demonstrated that not only MI did activate the alpha motoneurons, but that the skeletomotor discharge was also accompanied by recruitment of spindle afferents when the covert contraction was sufficiently strong. Furthermore, there is evidence of increasing EMG activity accompanying imagined mental effort (Boschker, 2001; Slade et al., 2002; Guillot et al., 2007). Finally, EMG activity has been observed not only in agonistic muscles, but also in antagonistic muscles, as a function of both the weight to be lifted (Bakker et al., 1996) and the muscle contraction type (Guillot et al., 2007). In this latter study, the authors found that the subliminal muscle responses during MI of concentric, isometric, and eccentric contractions typically mirrored the configuration of the EMG activity recorded during actual practice. These data support the hypothesis that muscle activity recorded during MI is not a general tonic activation but reflects the content of the specific motor command of the movement that is mentally rehearsed. According to Jeannerod (1994), an incomplete inhibition of the motor command could provide a valid explanation for these muscle discharges. So, it seems that imagined movements produce a qualitatively similar, but quantitatively smaller, drive to muscles compared with actual motor execution, thereby suggesting that a small part of the motor command is actually sent to the effectors during MI. Interestingly, Solodkin et al. (2004) argued that both the supplementary motor area and the lateral premotor cortex might also play a role in increasing muscle tone during MI – especially during KI. This speculation is plausible given that these brain regions have direct projections to spinal cord through the internal capsule, adjacent to the well-known corticospinal path originating from cM1 (Luppino et al., 1994; Morecraft et al., 2002).

Inconsistencies in the reports of concomitant EMG activity in the muscles participating in the movement during MI might be explained by differences in the experimental designs, as well as by the nature of the EMG recordings (Guillot et al., 2010). For instance, EMG activity might not be discernible due to the use of surface EMG electrodes and intramuscular electrodes should ideally be preferred, although intrusive and thus rarely used in MI experiments (except in the case of Gandevia et al., 1997). Analogously, the effect of the muscle contraction type, the intensity of the mental effort, and the intrinsic nature of MI may also contribute to understand why EMG activity was not systematically reported. Finally, with few exceptions, studies reporting a lack of EMG activity primarily investigated laboratory movements, whereas those experiments providing evidence of a muscle activity during imagery included more goal-related movements (e.g., skills in sport). Experimental data reporting muscle activity only in a part of the tested sample (Li et al., 2004a; Dickstein et al., 2005) lend support to the fact that muscle activity was not systematically discernible due to such confounding factors. Interestingly, the pattern of muscle activation has never been found to match the usual triphasic sequence generated during actual motor performance (Murphy et al., 2008). Furthermore, although traditional psychoneuromuscular theory (Carpenter, 1894) postulated that muscle activity recorded during MI might generate slight neuromuscular feedback, strong enough to improve subsequent motor performance through priming of the motor pathways, there is no direct evidence that muscle activation during MI is associated with improved motor performance. In other words, research has not yet demonstrated that the increase in muscle activity fully contributes to performance enhancement, but EMG recordings support that the motor command is actually prepared, and then blocked by inhibitory processes, during MI.

### Corticospinal facilitation patterns during MI

Transcranial magnetic stimulation activates neurons trans-synaptically (Rothwell, 1991) and allows the study of corticospinal facilitation (i.e., the level of excitability of the corticomotor pathway). Typically, the motor evoked potential (MEP) elicited by a suprathreshold TMS pulse delivered to cM1 is recorded at the peripheral level, using surface EMG. The development of repetitive pulse TMS protocols has allowed the study of intracortical facilitation and inhibition, and also helps to delineate excitatory and inhibitory interactions between different brain regions mediating motor control (Reis et al., 2008). In this section, we review some TMS studies suggesting that MI involves elaboration of motor command signals at the CNS level. We also discuss the extent to which MI activates the descending somatic motor pathways originating from pyramidal neurons and projecting toward the alpha motoneurons pool. Finally, we consider the recent hypotheses emerging from TMS findings regarding motor inhibition during MI.

CNS activity during MI and PP enables researchers to understand whether MI effectively involves motor commands processing. TMS studies provided converging evidence that MI increases the corticomotor excitability (Stinear, 2010). Excitability changes within motor cortical areas during MI, including reduced intracortical inhibition, are analogous to those observed during motor preparation and execution (Abbruzzese et al., 1999; Kumru et al., 2008). Intracortical inhibitory interneurons are known to play an essential role in the shaping of motor commands (Stinear and Byblow, 2003a). This phenomenon is thought to mirror an analogous motor activity at the cortical level during both tasks. Contrary to PP, intracortical inhibition of cM1 is not entirely removed during MI (Kumru et al., 2008). In general, whilst MI involves improved cortical facilitation and reduced intracortical inhibition, it does so with reduced amplitude compared to PP (Clark et al., 2004; Leonard and Tremblay, 2007). It has been suggested that the CNS manages to keep corticospinal facilitation below the motor threshold for activating the alpha motor neurons pool during MI (Stinear, 2010).

Does cortical activity during MI effectively reflect shaping of motor output – which may require motor inhibition? Firstly, corticospinal facilitation during MI is effector-specific according to MI content (Kasai et al., 1997; Stinear and Byblow, 2003a, 2004). The motor threshold in muscles involved into MI content is lower, while the amplitude of the subsequent MEP is higher (Facchini et al., 2002). By contrast, MEPs elicited in non-involved muscles remain unaffected (Facchini et al., 2002; Stinear and Byblow, 2003b; Quartarone et al., 2005). Furthermore, Leonard and Tremblay (2007) demonstrated that the muscle-specific pattern of corticospinal facilitation during MI was altered in aging populations, thus reflecting individual ability to shape motor output coding for isolated finger movements. Corticospinal facilitation during MI is also graded upon the extent to which the muscle is actually recruited during the corresponding motor performance (Yahagi and Kasai, 1998). Liang et al. (2007) further reported that corticospinal facilitation during MI of wrist flexions mirrored the synergic pattern of muscle activity produced by PP, a key feature of the motor command (see also Kasai et al., 1997, for results suggesting preservation of agonist-antagonist patterns of muscle activations during MI). Likewise, using TMS evoked muscle twitch, Li et al. (2004b) reported that MI of finger flexion preserved the unintended functional coupling in strength response between the digits, described as the “enslaving effect” (Zatsiorsky et al., 2000). Further, Facchini et al. (2002) observed that only MI of contralateral thumb abduction, but not ipsilateral, facilitated MEPs in the contralateral effector. This result supports the idea that MI reproduces the hemispheric specificity with regards to cM1 enrollment during imagination of lateralized movements. Finally, Fadiga et al. (1999) observed that MI of elbow flexion/extension increased MEPs in the biceps brachii merely during the timing portions of MI corresponding to the arm flexion, thus suggesting that corticospinal facilitation during MI matched the temporal features of physical performance.

To summarize, the accumulating evidence appears to suggest that the corticospinal facilitation is highly specific to the motor task. Increased corticomotor excitability during MI may not be a result of a general state of arousal due to execution of cognitive operations (Rossini et al., 1999; Clark et al., 2004; Fourkas et al., 2006), but instead due to the demands of the internal processing of motor output (Stinear, 2010).

As mentioned previously, most features of corticospinal facilitation during MI suggest internal elaboration of neural signals for muscle contractions. It is generally assumed that increased corticospinal facilitation during MI reflects analogous involvement of cM1 between MI and PP (Stinear, 2010). However, such increased corticomotor excitability may also reflect more general changes in the balance between excitatory and inhibitory impulses, which can occur at different stages of the somatic motor system. Understanding how the centrally shaped motor output is inhibited during MI initially requires further analysis of the extent to which CNS excitability changes (due to MI) affect the descending motor pathways. For instance, one may question whether corticospinal facilitation during MI involves the motor system at the spinal level. Yahagi et al. (1996) addressed this issue and observed that whilst MI of wrist flexions facilitated the MEPs in the flexor carpi radialis, no change was recorded in the H-reflex surface EMG traces evoked by electrical stimulation, thus revealing that corticospinal facilitation during MI occurred without any change in spinal excitability. This finding was replicated in several studies investigating changes in H-reflex during MI, in combination with TMS (Kasai et al., 1997; Hashimoto and Rothwell, 1999; Patuzzo et al., 2003). F-waves elicited by peripheral nerve transcutaneous electrical stimulation provide an objective measurement of spinal excitability, without interference from descending neural impulses of cerebral origin (e.g., cerebral spontaneous regulation of spinal reflexes). Rossini et al. (1999) reported a 9.8–14% increase in F-waves amplitude during MI of index and little finger abduction. These robust results challenge previous observations where no change in F-waves was recorded during MI of finger actions (Facchini et al., 2002; Stinear and Byblow, 2003a; Stinear et al., 2006). However, in the two movements investigated by Rossini et al. (1999), only MI of finger abduction elicited a slight increase by 5.9% in the TMS evoked MEP with no change recorded in the MEP latencies when compared to a non-motor mental activity (mental arithmetic). Rossini et al. (1999) stated that while MI may have increased spinal motoneuronal excitability, corticospinal facilitation during MI primarily reflected changes of cortical origin. Consequently, TMS data suggest the analogous involvement of cM1 into motor command processing during both MI and PP. Both tasks elicit excitability changes at the cortical level (Kasai et al., 1997; Abbruzzese et al., 1999; Patuzzo et al., 2003; Stinear and Byblow, 2004), whereas there is a paucity of robust TMS evidences of excitability changes at the spinal level. Consequently, TMS results indicate that inhibition during MI might intervene during the early stages of motor processing. Several neuroimaging findings support this contention, revealing that specific cortical and subcortical sites could contribute to prevent overt motor processing during MI (Lotze et al., 1999b; Kasess et al., 2008).

Challenges to these TMS-based accounts of motor inhibition during MI come from some EMG studies. For example, Bonnet et al. (1997) reported the sharp increase of both H- and T-reflexes during MI of strong foot pressure above a pedal. In this study, T-reflexes displayed a highly specific pattern of facilitation (i.e., lateralized and graded depending on the stimulated movement) which was not observed in H-reflexes facilitation. Bonnet et al. (1997) argued that MI elicited both spinal and spindle activation in the task-relevant corresponding effectors. This finding was replicated in studies reporting increased H-reflexes excitability during MI (Hale et al., 2003). As mentioned above, Gandevia et al. (1997) reported increased activity from spindle afferents using microneurographic recordings from the relevant muscles. The authors concluded that MI recruited both motor units and afferent spindles. Their results further demonstrated that, in some cases, the motor commands built up during MI might reach the muscle level and elicit neural feedback from muscle receptors. Gandevia et al. (1997) therefore stated that MI may consist of “*unintentional performance of (*…*) planned motor task*,” hence suggesting that somatic activity during MI might account for the observed effects of MI training on motor performance, through reinforcement of motor output conduction throughout the neuromuscular system (Gandevia, 1999).

Experimental studies also support the central elaboration of motor commands during MI. For example, both EMG and TMS findings support for the role of concurrent indirect information concerning motor inhibition during MI. Firstly, EMG data indicate that a residual motor command can be partially addressed to peripheral effectors during MI. Secondly, TMS findings suggest that the motor system keeps the facilitation of the corticomotor pathways below the motor threshold, in spite of a highly action-specific pattern of arousal. These two ways of understanding motor inhibition during MI could also represent different ways of analyzing a multimodal process: specific interactions between cerebral regions could result in the transmission of a residual motor command toward the descending volleys, whilst interactions between cerebral sites and/or spinal influences could keep corticospinal excitability below the motor threshold. For instance, a recent TMS finding asserts that the ipsilateral inferior parietal lobe might exert an inhibitory influence on cM1 during MI (Lebon et al., 2012).

## Motor Inhibition

Earlier in this paper, we showed that motor performance and MI are mediated by distinct neural networks, despite an extensive overlap between KI and PP. In particular, while mental operations of motor planning and programming are actually performed during MI, motor commands must be inhibited before being sent to peripheral effectors within the descending pathways. This inhibition process aims at preventing the performer from engaging in any movement during mental rehearsal. However, we previously underlined significant differences in cerebral activations elicited by MI and actual movement (Hanakawa et al., 2008), some of which being probably responsible of the inhibition of motor commands. Interestingly, Schwoebel et al. (2002) described unique behavior from a patient with bilateral parietal lesions. When imagining hand movements, the patient simultaneously executed the imagined motor act but without being aware of the movements. Surprisingly, these movements were also significantly more accurate than volitional movements. The findings from this clinical case study are consistent with previous accounts suggesting that MI may normally involve the inhibition of movements.

Most studies dealing with MI generally give information about the inhibition of motor commands by providing EMG recordings. This is a reliable means to ensure that brain activation recorded during MI actually comes from mental representation and not from potential motor activity that could have accompanied the mental task. Thus, EMG recordings during MI should be comparable to those that occur during rest. However, a common challenge is that across most investigations, MI and rest conditions are rarely accurately compared. Guillot et al. (2007) observed significant increased pattern of EMG activity in all muscles of the arm, forearm, and even shoulder during MI of forearm flexion, when compared to the rest condition, while goniometric data did not reveal any movement. The magnitude of this activation was correlated with the mental effort required to imagine lifting a weight. Indeed, MI of heavy concentric contraction (80% of the best mark) resulted in greater pattern of EMG activity than during MI of light concentric condition (50% of the best mark). The intensity of the imagined contraction was thus paralleled by the magnitude of the subliminal EMG activity, thereby highlighting a close link between the central nervous system and the periphery during MI. Bakker et al. (1996) and Boschker (2001) had previously found that mentally lifting a 9 kg dumbbell resulted in a larger EMG activity than lifting 4 kg 1/2. Jeannerod (1994) and Bonnet et al. (1997) attributed changes in EMG activity during MI to an incomplete inhibition of the motor command. This hypothesis was emphasized by differential muscle activity associated with the contraction type. Interestingly, different types of mentally rehearsed contractions elicited specific changes in EMG activity that closely corresponded to those observed during actual contraction.

The preceding evidence shows that MI might recruit the same movement pattern as the actual motor command, although at subliminal intensity, hence involving the same neural substrate. Thus, EMG activity during MI seems to mirror that observed during actual motor execution. Importantly, this was not a tonic non-specific activity as the patterns of EMG distinguished among the different types of contraction to the same extent as actual execution would have done.

Arising from the argument so far, two questions need to be addressed. First, how useful is this specific residual motor command? Second, what are the neural substrates of partial inhibition of the motor command? One of the most plausible outcomes is that sensory afferent information provided to the CNS should serve as feedback in the hypothesis of a forthcoming actual movement. Secondly, the cerebellum might be involved in the inhibition of movement execution during MI (Lotze et al., 1999b).

The question of inhibiting movement execution, after the motor commands have been prepared, has often been asked (e.g., see de Jong et al., 1990). Motor inhibition is usually tested with the “Go/No-Go” paradigm. Here, participants are requested to give a motor response when a specific stimulus is presented and to withhold the response occasionally when another stimulus is triggered. Reaction time to the “Go” signal is recorded, thus facilitating the study of how the motor system inhibits the response when the “No-Go” signal is randomly given. Typically, Go/No-Go paradigms elicit a race between response activation and response inhibition processes. de Jong et al. (1990) postulated the existence of two inhibitory mechanisms: inhibition of central activation processes and inhibition of transmission of motor commands from central to peripheral structures. Unfortunately, the way in which these inhibitory processes work may not directly be applied to MI. For example, the processes of motor command inhibition during MI may not work exactly as during those elicited by the Go/No-Go paradigms because, in the latter, participants do not know in advance whether they will have to act or to inhibit action. By contrast, when participants are requested to mentally represent an action, they implicitly know that they will restrict their cerebral activity to covert movement only. Thus, motor command inhibition should be integrated into the process of movement preparation through motor representation. As postulated by de Jong et al. (1990), a possible mechanism for response inhibition (which could be applied to MI), involves the inhibition of central response activation processes. Thus, response initiation might be inhibited by preventing central response activation from reaching the targeted muscles. In this way, the interruption of an already initiated response can be achieved by discontinuing the output from central to peripheral motor structures. This speculation was experimentally attested by large fronto-central positivity when the response was successfully inhibited (de Jong et al., 1990). The inhibitory mechanisms are effective before sending the information, elaborated within the associate cortices, to the primary motor cortex. Especially, this inhibition may originate from the prefrontal cortical areas associated with limbic structures and cingulate cortices (the behavioral inhibition system early postulated by Gray, 1990). This behavioral system has other connections with the parietal associative cortices involved in No-Go performance (Watanabe et al., 2002). However, response inhibition could also come from active mechanisms at different subcortical levels including the spinal cord (Bonnet et al., 1997), the brainstem, and the cerebellum (Lotze et al., 1999b). A particular example is when the programming of a movement is not entirely well-adapted to its expected goal and requires changing one or several parameters, such as movement direction or amplitude. In this case, we do not need to fully inhibit actual performance but only to better adapt the programming of movement to the environmental constraints under which it occurs. Thus, a flexible central inhibitory mechanism may become crucial when selective motor inhibition is required. Many results from neuroimaging research suggest that the right inferior frontal gyrus is integrated within a fronto-basal-ganglia network (Aron et al., 2007), which could intercept the “Go” process and stop the motor responses (Lenartowicz et al., 2011). This function is also consistent with a role in reprogramming of action plans, which may comprise inhibition, and its activity can be triggered through automated, bottom-up processing.

If central inhibition processes do not succeed in preventing central motor outflow, the overt response can be inhibited by preventing the transmission of motor commands to peripheral motor structures. This possibility is consistent with the hypothesis that motor commands could be inhibited at any time (de Jong et al., 1990). In other words, the inhibition of overt movements may still be achieved by means of peripheral mechanisms. By contrast with central inhibitory mechanisms, peripheral inhibitory mechanisms may be useful only when actions have to be inhibited or interrupted unselectively. Brunia (2003) proposed that there are several inhibitory mechanisms at work in the periphery of the motor system, all depending upon activity of local propriospinal interneurons, situated at the same or neighboring segments of the spinal cord as that of the motoneurons of agonist or synergist muscles. Normally, corticospinal fibers contact alpha motoneurons mono-synaptically. However, while this organization works for hands and fingers, it probably does not hold for other body segments. To result in a movement, the influence of propriospinal neurons upon motoneurons has to be excitatory. The intrinsic organization of the spinal cord enables movement production including several inhibitory systems such as the short feedback system from the Renshaw cells or the reciprocal inhibition reflex system. These processes are beyond the scope of the present paper, however. We thus hypothesize that motor inhibition during MI is mainly related to the *central* but not to the peripheral system.

Logan (1983) conducted several studies on the degree to which people inhibit the thoughts that underlie their actions when they inhibit action. Participants were requested to make category and rhyme judgments about words and were given stop signals that required them to inhibit the actions they executed to express their judgments. They pressed one key if the word was a member of the category or rhymed with a target, and pressed another key if the word was not a member of the category or did not rhyme. If a stop signal occurred, they were supposed to inhibit the response. These researchers then presented the materials again to test participants’ memory for words whose responses were inhibited, and used recognition memory judgments: they presented both words for which that they had made or not judgments about, and asked them to indicate whether words had been presented before. Subsequently, Logan (1985) used repetition priming to test memory by presenting similar kind of words, and then asking whether response time was faster for old words than for new ones. Both studies revealed that thoughts went on to completion when actions were inhibited, suggesting that mental activity was independent from the motor response. As far as we consider MI, mental activity is directly related to action, thus suggesting two related processes differing from the relationships highlighted by Logan (1983, 1985).

As previously mentioned, de Jong et al. (1990) described two inhibitory mechanisms that could work to withdraw actual motor command: inhibition of central activation and inhibition of transmission of motor commands from central to peripheral structures. If we consider that motor planning and programming are central processes, we can assume that these are common to actual execution and MI. Hence, the difference between these two behaviors would be the existence of an active process of motor command inhibition. Now, the question of how motor command inhibition is neurally implemented remained unresolved. But what exactly does research tell us about motor command inhibition and how can this knowledge be applied to MI? Most experiments on motor command inhibition were conducted using stop signal paradigms, early formalized by Logan and Cowan (1984). The key component of response inhibition depends upon the relative finishing time between Go and No-Go operations. In other words, the Go response is inhibited by the activation of a stop-process. The major difference between inhibition of motor command in the context of action execution vs. MI is mainly related to *uncertainty*. Uncertainty is emphasized in an updated model by Boucher et al. (2007). A Go response may also be inhibited by the preparation of an alternative go response. In this case, response inhibition would depend on the relative finishing time of the primary-task response and the alternative response (Verbruggen and Logan, 2009). In both models of response inhibition, the participant does not know in advance whether he/she will have to withhold the motor command in the Go/No-Go paradigm. When requested to mentally imagine a movement, the participant is clearly aware that no command should be transferred to peripheral effectors when he/she is requested to mentally imagine the action. Inhibition does not rely on the same process in both conditions. In the stop paradigm, response inhibition depends on triggering No-Go signal. Once the Go or No-Go stimulus is perceived, the participant should decide to act or to inhibit action, taking into account the information provided. Although action vs. inhibition could simultaneously be prepared as an alternative response, this is an all or nothing process. There is no such uncertainty during MI. However, the inhibition of movement may be total or partial and may also take even several intermediate degrees. In other words, the participant could nevertheless accompany the mental representation by residual execution, e.g., miming partially some significant steps of execution or movement rhythm. The other main difference is related to the *time course* of these processes. On the basis of event-related brain potentials, EMG recordings, and continuous behavioral response measures, experimental data from de Jong et al. (1990) evidenced that responses could be interrupted at any time. Thus, actual movement is inhibited as early as the stop signal is triggered whereas MI could accept several conditions from no movement at all until residual movements related to actual movement that accompany and facilitate mental representation. This may explain why MI could also keep some elements of motor execution during mental representation. However, and with reference to the casual definition of MI, we should wonder whether we could still call this process “imagery” when associated with residual parts of movements.

Finally, central inhibition processes are well-summarized by Garavan et al. (2002) who postulated two main neural networks mediating inhibition. Right dorsolateral prefrontal and right inferior parietal areas are associated with response inhibition while a region of the cingulate cortex is involved in “difficult” inhibitions. Left dorsolateral prefrontal cortex was activated when subjects adjusted their ongoing behavior in response to an error or to unexpected changes in the environment. With regard to selective inhibitory mechanisms, it is therefore not surprising that residual muscle activity remains observable during MI, through EMG recordings, as described in preceding paragraphs. This is compatible with both hypotheses previously described: On the one hand, motor commands could be inhibited at any time and in different ways. On the other hand, MI could be dependent upon the central process of inhibition only. Overall, we should point out again that inhibition of the actual command, based on Go/No-Go designs, does not exactly correspond to that during MI, mainly because this inhibition is not under the control of decision making under time pressure. Therefore, a specific cerebral organization might control motor commands inhibition during MI, nevertheless sharing most features of the central processes inhibition we previously described.

In summary, the issue of inhibiting motor performance can be explained by two theoretical models. We first assume that MI results in a subliminal activity of the motor system. As postulated by Jeannerod and Frak (1999), the motor system is involved not only in the production of movements, but also in the mental representation of action. The authors extended its function to the process of learning by observation, even until understanding the behavior of others. Therefore, if we consider MI as a subliminal motor command, it will not cause muscle activity and there is no need for active inhibition process. The functional similarity between actual movement and MI comes from the identity of the motor structures that are believed to control them. Thus, the only difference between actual execution and its mental representation would be the degree of mobilization of motor commands: the preparatory phase would be common to actual action, its mental representation, and the consequences of action both in terms of sensations generated and knowledge of result (did the action reach its goal?). In this regard, Macuga and Frey (2012) recently postulated that the neural representations of observed, imagined, and imitated actions were dissociable and hierarchically organized. The differential activity among these three conditions favored an alternative hierarchical model in which these behaviors rely on partially independent mechanisms. This result might challenge the hypothesis of complete similarity between actual movement and MI, and therefore favor the second hypothesis.

More pragmatically, it is easy to experience motor representation in association with movements or sequences of movements, more or less related to the imagined action and supposed to accompany and facilitate MI. One of the most remarkable examples is when observing some elite athletes during the preparation phase, just before competing. For example, some skiers prepare for races by closing their eyes and mentally rehearsing the course that they are about to traverse (Louis et al., 2012). Although they probably do not experience the entire course, some portions are nevertheless mentally rehearsed using symbolic limb movements – which enable them to mime the represented action. These movements of arms and hands symbolize the turns and the timing at which these should be done. Finally, as revealed by Lorey et al. (2009), we are all familiar with pictures of athletes moving while imagining their subsequent performance during pre-performance routines. Theoretically, such phenomena raise an interesting question. Strictly speaking, is it valid to describe MI performed with associated movement as “MI”? Although this question goes beyond the scope of the present paper, it is important to remember that specific subliminal muscle activity is detectable during MI of any given movement (Guillot et al., 2007). We would also point out that the theoretical mechanisms we described above have the potential to explain how inhibition works during MI. In particular, the inhibition process could occur at *every stage* of the represented action: complete inhibition during MI would mean that actual movement is entirely removed from MI (this corresponds to the usual definition which is often given, “*MI is* *the mental representation of an action without any overt movement*”). The hypothesis of partial motor inhibition could also be invoked, and as previously mentioned, we may combine movements to their mental representation, even if they are only partially outlined.

How does MI affect motor commands? Performing MI might activate somatic and autonomic motor commands differently. From the intention to act, direct voluntary commands are normally transmitted through the pyramidal tract to elicit movement. The process of an incomplete inhibition that accompanies MI may be viewed at organizing peripheral effectors during the preparation phase of the forthcoming actual execution. Duclos et al. (2008) provided evidence of anticipatory changes in patterns of human motoneurons discharge during motor preparation. This may also be observed during MI. Conversely, Bonnet et al.’s (1997) view is that MI should be compared to action, rather than to motor preparation, hence considering MI as the intention to avoid movement execution, although MI might be more closely related to pre-executive processes of a movement than its actual execution itself (Hanakawa et al., 2008). Michelon et al. (2006) claimed that the MI process does not necessarily require a motor simulation which would integrate the mapping of the effector-specific commands required to achieve the movement. This would be a quite different MI as that followed by actual execution. The close relationship (temporal, structural) between MI and actual execution appears to favor an upstream organization of inhibition, implying the behavioral inhibition system. This would also explain why the most automated parts of movement commands are not inhibited, as they are controlled at the subcortical level. Nevertheless, Bonnet et al. (1997) and Jeannerod (2006) stated that the inhibitory mechanisms may also be localized downstream of the motor cortex, perhaps at the spinal cord, or brainstem level. In addition, Lotze et al. (1999b) postulated that the posterior cerebellum might also play a crucial role in the inhibition of the motor command. There are probably several systems and processes of motor inhibition, coordinated at different levels of the central nervous system from the premotor cortex to the spinal cord. The question of a selective inhibition remains to be asked. It could explain the subliminal muscle activity and even the somatic commands addressed to the low levels of the CNS (e.g., controlling postural regulations).

Several experimental data provide evidence of incomplete inhibition of the motor commands addressed to the different effectors. This finding should be used in the field of clinical rehabilitation (whatever the nature of the neurological damage either peripheral or central), MI use is known to benefit to functional recovery (e.g., Braun et al., 2006; Zimmermann-Schlatter et al., 2008). Further research should also investigate the processes of somatic and autonomic motor commands inhibition during MI. So far, two different mechanisms are thought being involved in the inhibition of a voluntary action. The first is related to central programming processes whereas the second is responsible for central motor command transmission from central structures to peripheral effectors (de Jong et al., 1990). As early hypothesized by Jeannerod (1994), the neural commands for muscle contractions may be blocked at some level of the motor system by active inhibitory mechanisms. This purpose is, however, associated with incomplete inhibition of the motor command that would provide a consistent explanation for the recording of muscle activity during MI.

## Clinical Experiments

Earlier in this paper, we reviewed evidence that motor commands are involved during MI before being inhibited. In this section, we discuss the extent to which clinical data obtained from patients with stroke, PD and also from amputees and those with SCI provide further evidence on this issue. Firstly, analyzing central activity during MI in patients suffering from central nervous system disorders reinforces the postulate of analogous central processing between MI and motor performance (Table 2). MI interventions can thus be used to improve motor processing after various cases of neurological disorders (Sharma et al., 2006; Dickstein and Deutsch, 2007). Secondly, motor impairments due to neurological diseases are reflected by changes in MI ability. This conclusion holds in most clinical populations, and therefore validates the assumption that MI reproduces actual motor performance states at the CNS level – yet without going to completion, hence the hypothesis of motor inhibition. Eventually, changes in MI ability after neurological disorder provide new perspectives to the study of motor command inhibition during MI.

**Table 2:** **Studies looking at the motor command during motor imagery**.

Study	Sample of patients and controls	Methodology	Result regarding MI ability changes and potential implications for understanding motor command processing and/or subsequent motor inhibition
Alkadhi et al. (2005)	SCI (*n* = 8) Controls (*n* = 8)	fMRI	MI recruited the neural networks subserving MI and actual movements in healthy controls. Primary motor cortex activity during MI in patients was activated to the same extent than during actual practice in healthy controls, suggesting weakened inhibition
Cramer et al. (2005)	SCI (*n* = 12) Controls (*n* = 12)	fMRI	No task modulation in cerebral activity between MI and PP. Reduced activation volumes in the primary sensorimotor cortex and increased activity within the primary sensorimotor cortex during MI reflect brain function changes after SCI
Gustin et al. (2010)	SCI (*n* = 11) Controls (*n* = 19)	fMRI	Contrary to controls, MI elicited activity within the primary motor area and several brain regions included in the pain neuromatrix. Activity correlated to pain perception during MI
Hotz-Boendermaker et al. (2008)	SCI (*n* = 9) Controls (*n* = 12)	fMRI	Cerebral activity during attempted and imagined movement supports motor program preservation. Recruitment of additional brain regions during MI (compared to healthy controls) reflects altered sensorimotor integration
Lacourse et al. (1999)	SCI (*n* = 19) Controls (*n* = 10)	EEG	Isomorphic electrophysiological correlates during MI and attempted execution in SCI patients, but not in healthy controls. Weakened inhibitory mechanisms as a consequence of SCI, due to deafferentation
Olsson (2012)	SCI (*n* = 1) Controls (*n* = 8)	fMRI	Changes in MI ability according to the remaining capabilities of the motor system
Battaglia et al. (2006)	Stroke (*n* = 8) Controls (*n* = 10)	TMS	Reduced corticospinal facilitation supporting that unilateral stroke patients have lateralized MI deficits
Daprati et al. (2010)	Stroke (*n* = 32) Controls (*n* = 12)	Mental rotation	Impaired MI ability. Patients may have developed MI strategies independently from the actual state of the motor system
Decety and Boisson (1990)	SCI (*n* = 4) Brain injury (*n* = 6)	Mental chronometry	Contrary to SCI patients, stroke patients presented longer MI times when engaging the paralyzed upper/lower limb, comparing to MI of actions with unaffected limbs. For movements that could be physically executed, patients achieved the temporal congruence between MI and executed actions
Dettmers et al. (2012)	Stroke (*n* = 31) Tetraparetic (*n* = 10)	Mental chronometry KVIQ VMIQ	MI ability is impaired on the affected side of the lesion, specifically after stroke eliciting deafferentation. In both clinical populations, the features of MI ability reflect the actual state of the motor system
Gonzalez et al. (2005)	Stroke (*n* = 11) Controls (*n* = 11)	Mental chronometry	Higher MI and PP times in patients who recovered from stroke than in healthy controls
Kagerer et al. (1998)	Brain injury (*n* = 4) Controls (*n* = 4)	Mental chronometry	Patients exhibited longer MI and PP times than for actions involving the more affected side with preserved temporal congruence between MI and PP
Kimberley et al. (2006)	Stroke (*n* = 10) Controls (*n* = 10)	fMRI	Cerebral activity during MI reflects the ipsilateral control of the stroke-affected hand, a common plastic brain change after lateralized stroke lesions
Liepert et al. (2012)	Stroke (*n* = 20)	TMS Mental chronometry	MI ability impaired for movements involving the stroke-affected hand, but only in patients suffering from a somatosensory brain lesion compared to patients with “*pure motor strokes*”
Malouin et al. (2008)	Stroke (*n* = 32) Controls (*n* = 32)	KVIQ	Patients obtained better scores when MI concerned the unaffected side of the lesion, but only for MI of lower limb actions
Sabate et al. (2004)	Stroke (*n* = 9) Controls (*n* = 10)	Mental chronometry	Decreased movement velocity during PP also observed during MI. The hemispheric-dependent effects of lateralized stroke on the actual motor performance of each hand (affected/non-affected) was reproduced during MI
Sabate et al. (2007)	Stroke (*n* = 33) Controls (*n* = 18)	Mental chronometry	Strong correlation between MI and PP times after stroke. Mismatches between MI and PP times support that changes in MI ability reflect the actual state of the motor system	PD (*n* = 8)
Schwoebel et al. (2002)	Stroke (*n* = 1)	Motor tasks Mental rotation	A patient with bilateral parietal brain lesion fully executed the mentally rehearsed actions. Inhibition during MI was impaired, presumably due to disturbances within a fronto-parietal circuit mediating motor inhibition
Sirigu et al. (1995)	Stroke (*n* = 1)	Mental chronometry	Temporal parameters of MI predicted that of PP in a variety of situations, altogether reflecting hypokinesia after unilateral stroke
Sharma et al. (2009a)	Stroke (*n* = 8) Controls (*n* = 13)	fMRI	Abnormal functional connectivity patterns within the motor network during MI correlated with motor outcome after stroke recovery
Sharma et al. (2009b)	Stroke (*n* = 20) Controls (*n* = 17)	fMRI	During MI of the affected hand, activation of the anterior subdivision of cM1 was similar to that during PP, and activity of the ipsilesional posterior subdivision of M1 correlated with motor performance. The result support that MI reveals the actual state of the motor system after stroke
Stinear et al. (2007)	Stroke (*n* = 12) Controls (*n* = 8)	Mental chronometry TMS	Absence of corticospinal facilitation during MI in the stroke-affected hand
Szameitat et al. (2012)	Stroke (*n* = 5) Controls (*n* = 21)	fMRI	Cortical activations during MI resemble that during attempted overt execution within sensorimotor and premotor cortices. Potential analogous involvement of the sensorimotor system in the two tasks
Vromen et al. (2011)	Stroke (*n* = 21)	Mental rotation	Stroke patients (*n* = 20) without spatial neglect outperformed a patient (*n* = 1) with spatial neglect during a visual mental rotation task involving the upper limb
Wu et al. (2010)	Stroke (*n* = 18)	Mental chronometry	Longer times required to imagine upper limb actions involving stroke-affected effectors
Cohen et al. (2011)	PD (*n* = 24) Controls (*n* = 10)	Mental chronometry	Temporal discrepancies between times required to imagine and actually walk through a narrow doorway characterized PD patients with freezing of gait syndrome
Cunnington et al. (1997)	PD (*n* = 14) Controls (*n* = 10)	EEG	Impaired motor preparation, while potentials associated with motor execution seemed relatively preserved
Cunnington et al. (2001)	PD (*n* = 6) Controls (*n* = 3)	PET	Reduced pre-SMA activation and compensatory brain activity during MI altogether characterized the motor deficit in PD patients
Dominey et al. (1995)	PD (*n* = 7) Controls (*n* = 7)	Mental chronometry	Asymmetrical slowing of MI times according to the affected side in lateralized PD patients, hence supporting that MI and PP shared common neural structures
Helmich et al. (2007)	PD (*n* = 19)	fMRI	MI involving the affected side in lateralized PD patients recruited additional cognitive resources compared to MI involving the unaffected side
Helmich et al. (2012)	PD (*n* = 38) Controls (*n* = 19)	fMRI	Distinct sensorimotor processing at the subcortical level during MI characterized patients with and without resting state tremor
Heremans et al. (2011)	PD (*n* = 14) Controls (*n* = 14)	MI questionnaires Mental chronometry	MI ability was preserved in PD patients, but was performed more slowly than in healthy controls
Kuhn et al. (2006)	PD (*n* = 8)	EEG	Analogous contribution of subthalamic nucleus to feedforward organization during MI and PP of wrist actions. Electrophysiological correlates of MI within these structures support its role in sensory feedback integration for overt motor and postural regulations after PD
Samuel et al. (2001)	PD (*n* = 6) Controls (*n* = 6)	PET	MI yielded decreased activity in frontal areas (dorsolateral frontal cortex), hence reflecting impaired motor preparation in PD patients as compared to healthy controls
Thobois et al. (2000)	PD (*n* = 8) Controls (*n* = 8)	PET	MI elicited reduced activations for movement with the affected side in lateralized PD patients. MI of the unaffected side was impaired, but to a lesser extent
Thobois et al. (2002)	PD (*n* = 7)	PET	Subthalamic nucleus stimulation analogously improved cerebral activity during MI and PP
Diers et al. (2010)	Amputees (*n* = 14) Controls (*n* = 9)	fMRI	MI activated different neural substrates depending on whether amputee patients experienced phantom limb pain or not. MI activated the contralateral primary sensorimotor cortex only in non-pain patients
MacIver et al. (2008)	Amputees (*n* = 13) Controls (*n* = 6)	fMRI	MI training elicited reversed sensorimotor plasticity in amputee that corresponded to decreased phantom limb pain symptoms
Marconi et al. (2007)	Amputees (*n* = 8) Controls (*n* = 9)	TMS	MI mirrored sensorimotor reorganizations in the patients. Upper/lower limb inhibitory relationships within cM1 might be removed after amputation
Nico et al. (2004)	Amputees (*n* = 16) Controls (*n* = 7)	Mental rotation task	MI was affected by amputation in patients as compared to healthy controls. Selective MI impairments were observed according to whether amputation affected the dominant/non-dominant limb
Raffin et al. (2012b)	Amputees (*n* = 14)	fMRI	Partially overlapping, albeit non-identical, neural networks mediating MI, and attempted physical practice with the phantom limb

### Stroke

Motor imagery ability seems preserved in most cases following stroke (Johnson et al., 2002; Sabate et al., 2007; Malouin et al., 2008). However, MI accuracy impairments were described in stroke and brain injured patients. These changes support the assumption that, during MI, the CNS reproduces a state of actual motor processing. Indeed, MI ability changes mirror those observed during actual motor performance. For instance, the time required to perform mental rehearsal of actions involving impaired limbs increased compared to that of actions performed with spared effectors (e.g., Decety and Boisson, 1990; Sirigu et al., 1995; Wu et al., 2010; Dettmers et al., 2012). Stroke patients also reported decreased imagery vividness during imagination of movements performed with the affected side in the case of lateralized brain lesions (Malouin et al., 2008). Malouin et al. (2004) suggested that temporal uncoupling between MI and PP could also occur during mental simulation of actions involving the stroke-unaffected side. Nonetheless, most findings in stroke patients support the assumption that MI mirrors motor impairments resulting from cerebral damage (Table 2). Inconsistencies regarding MI ability changes after stroke (i.e., specific or non-specific to the motor impairment) might account for the nature and localization of the stroke lesion (Liepert et al., 2012).

Imagery studies on stroke patients are largely consistent with results obtained from studies in healthy subjects – showing that MI is a dynamic state of motor processing, reproducing the features of CNS activity in a similar way to that during actual motor performance. Examining MI ability after brain lesions can thus contribute to understanding of the neural processes mediating actual motor performance. Sirigu et al. (1996) reported that mismatching between MI and PP times characterized the parietal brain lesion in stroke patients. The authors inferred that the parietal cortex might play a key role in elaboration of movement representation during motor preparation. More recently, Stinear et al. (2007) found that right hemisphere stroke patients *overestimated* MI duration as compared to PP, while left hemisphere stroke patients achieved more accurate temporal congruence between actual and imagined time. These data support hemispheric specificity with regard to internal generation of the temporal parameters of actual execution. As the temporal characteristics of MI are also affected by lesions, these results corroborate previous findings with regard to the neural substrates mediating movement preparation during MI.

To summarize, studies of MI ability in stroke patients show that MI and PP share common neural substrates and involve similar motor commands. But to what extent do imagery data obtained from stroke patients shed light on the issue of motor inhibition during MI? As MI ability is usually preserved following trauma to the nervous system, one should assume that inhibition remain possible even after cerebral damage. As several studies have reported that MI ability is preserved after brain damage affecting both cortical and subcortical motor networks, we may postulate that cerebral structures mediating motor control do not play a critical inhibitory role. This assumption would be congruent with some TMS findings assuming that there is no specific suppressive mechanism occurring at the brain level to inhibit motor output during MI, which would rather be caused by an incomplete level of CNS facilitation during MI (i.e., the level of excitability would not reach the motor threshold during MI, contrary to during PP). Nonetheless, Schwoebel et al. (2002) reported the case of a stroke hemiparetic patient with bilateral parietal brain lesions around the primary somatosensory cortex, who fully executed the “imagined” actions. As the patient performed more efficiently the demanded motor act during MI than during PP, authors argued that overt movements during MI reflected overt processing of the forward models for overt actions. These would be preserved and recalled during MI. Further, the authors nicely demonstrated that sensory integration, mediated by the primary somatosensory cortex, distinguished between overt and covert performance. Impaired sensory integration due to brain lesion thus explained both accurate actual executions during MI and altered voluntary motor performance during PP. Critically, this case report indicated that the motor command was effectively built up during MI, and may normally be actively inhibited throughout motor processing by specific interactions between sensorimotor brain regions. While the precise mechanisms underlying this effect remain unclear, the hypothesis that sensory feedback integration may be a key component for efficient motor suppression during MI is supported by neuroimaging findings (Solodkin et al., 2004; Alkadhi et al., 2005).

### Parkinson’s disease

MI ability changes in patients suffering from PD are consistent with those observed in stroke patients. Central processing during MI is selective according to the limbs affected by PD and mirrors the actual motor impairment (Dominey et al., 1995; Helmich et al., 2007), even though MI of non-affected body regions may also be disturbed to a lesser extent (Thobois et al., 2000). In spite of basal ganglia dysfunction, MI ability is preserved in early and mid-stage PD patients (Heremans et al., 2011). However, several neuroimaging studies have discovered abnormal brain activation patterns during MI in PD patients as compared to healthy controls (Cunnington et al., 1997; Samuel et al., 2001). Specifically, reduced premotor and sensorimotor activations, as well decreased cerebellum activation during MI were reported (Thobois et al., 2000; Cunnington et al., 2001; Samuel et al., 2001). Compensatory activations occurring during MI of actions involving affected effectors were also reported in PD patients, which could reflect the actual motor deficit (Cunnington et al., 2001; Thobois et al., 2002; Helmich et al., 2007). Of great interest, however, is the finding that subthalamic nucleus electrical stimulation enabled reduction of compensatory activations during MI in PD patients (Thobois et al., 2002). These data provide meaningful evidence that elaboration of motor command *does*, in fact, occur during MI after PD, as the central MI activity seems to mirror the effect of PD on actual motor performance (Cunnington et al., 1997; Samuel et al., 2001; Tremblay et al., 2008). Spontaneous eye movements occur during MI and resemble those occurring during PP (Heremans et al., 2008). Heremans et al. (2012) observed that external visual cueing even reinforced MI accuracy in PD patients, thus confirming that the central processing of somatic motor signals was an intrinsic component of the MI experience. Finally, several studies evidenced that PD patients may benefit from MI training in the rehabilitation of motor disorders, which suggests skill transfer from MI to PP with regards to actual motor processing (Mannix et al., 1999; Lim et al., 2006; Braun et al., 2011). Tamir et al. (2007) reported the adjunctive benefits of MI training in motor rehabilitation following PD. MI practice contributed to reduce bradykinesia (Subramanian et al., 2011). PD typically refers to basal ganglia dysfunction. These structures are known to play a role in overt motor performance inhibition in healthy subjects, through specific neural interactions with cM1 during the early stages of motor processing (Stinear et al., 2009). However, it remains unclear whether these structures also participate in motor inhibition during MI, but recent findings suggest that PD patients lose the ability to elicit corticospinal facilitation during MI (Tremblay et al., 2008). The authors assumed that the patients failed to involve the motor system during MI. Nonetheless as the MI ability seems preserved and to elicit sensorimotor activity at the brain level in most neuroimaging studies, it can be hypothesized that changes in basal ganglia activity during MI could increase inhibitory interactions during MI. This postulate remains a working hypothesis awaiting experimental proofs.

### Amputees

Consistent reorganizations of the cortical sensorimotor map occur after limb amputation, due to neuroplasticity (i.e., the capability of synapses to adapt their structure and function in response to environmental and behavioral demand). The expansion within primary sensorimotor cortices of the cortical surface corresponding to unaffected body parts toward the adjacent deafferented and deefferented areas (i.e., corresponding to the missing limb) is now well-established (Knecht et al., 1996; Pascual-Leone et al., 1996; Karl et al., 2001; Ramachandran et al., 2010). Nonetheless, the motor system preserves the ability to process central commands controlling the missing limb following amputation, as suggested in several neuroimaging studies reporting similar sensorimotor activations during actions performed either with the phantom limb or with the contralateral unaffected one (Ersland et al., 1996; Roux et al., 2001, 2003). EMG recordings at the level of the stump revealed specific motor commands suggesting preservation of motor programs controlling the amputated limb (Reilly et al., 2006). Are motor commands processed during MI of actions with the phantom limb? Recent findings suggest that the neural networks mediating MI and PP of actions with the phantom limb in amputee patients consistently overlap, in spite of significant differences (Raffin et al., 2012b). Most findings regarding imagery in these patients are derived from studies investigating the therapeutic management of phantom limb pain (i.e., a frequent disabling consequence following amputation; Shukla et al., 1982; Ehde et al., 2000). A causal relationship was found between sensorimotor reorganizations and phantom limb pain (Flor et al., 1995; MacIver et al., 2008). Expansion of homunculus regions corresponding to spared body parts toward deafferented areas (e.g., hand region “invaded” by regions corresponding to face body parts), in both primary somatosensory and motor cortices, characterize phantom limb pain patients as compared to pain-free amputees (Lotze et al., 1999a, 2001; Karl et al., 2001). Interestingly, involving the motor system into overt motor processing (i.e., attempted executed actions) with the phantom limb (e.g., using visual feedback) tends to produce pain relief (Chan et al., 2007; Mercier and Sirigu, 2009). An interesting practical implication of this finding is that if MI reproduces overt motor processing states at the CNS level, then MI *training* might also be efficient in the management of neuropathic pain after amputation. MacIver et al. (2008) investigated the effects of a 6-week MI training program on phantom limb pain. fMRI scanning sessions were performed before and after the experimental intervention. MI training significantly reduced pain symptoms. fMRI investigations highlighted a reversed plasticity as compared to plastic changes due to amputation observed in the patients during the pretest. These results promote the therapeutic relevance of MI in the rehabilitation of pain disorders, presumably due to actual motor processing at the CNS level. Indeed, change in the cortical representation of a body segment is usually achieved through repetitive motor practice (Wolf et al., 2002). In the study by MacIver et al. (2008), MI elicited reversed plasticity, hence supporting the assumption that amputees are able to process motor commands during MI, even for actions with their injured limb. In this regard, however, the fact that phantom limb pain patients and those with and non-phantom limb pain present *different* brain responses to MI is of special interest (Diers et al., 2010). Only non-pain patients activated the contralateral primary sensorimotor cortex during MI. Some authors have reported that neuropathic pain after amputation could be due to the mismatching between motor output and sensory feedback (Mayer et al., 2008), in agreement with the model of pathological pain by Harris (1999). MI might therefore reproduce a pathological state where the motor system fails to elicit the primary sensorimotor activity demand of the cognitive task. These findings concur with a large body of literature supporting the role of differential neuroplasticity in the generation of pain symptoms, which seems reproduced during MI. Further, it can be assumed that amputees no longer require to inhibit the motor command during MI of actions involving the phantom, as no muscle activity could occur (a similar observation can be made in SCI patients – see below). If MI *does* involve active motor suppression at the CNS level, these mechanisms will no longer be relevant after amputation and might potentially be reshaped by the emergence of a new body schema, prompting considerable neural reorganization. Challenging considerations to this approach comes from the fact that amputee patients usually preserve a perceptual representation of their missing limb. Further, no study yet reported weakened inhibition during MI after amputation and no EMG activity was recorded at the level of the stump (Raffin et al., 2012a). This is an interesting perspective for future studies: If there is no changes in the ability to inhibit the motor commands during MI, conclusions regarding the neural underpinnings of motor suppression during MI might be drawn.

### SCI patients

Motor activity during MI can be inferred from the effects of MI practice in neuropathic pain SCI patients. Gustin et al. (2008) observed that MI increased neuropathic pain intensity in six SCI patients out of seven during mental rehearsal of actions involving infra-lesional effectors. Gustin et al. (2010) delineated the neural substrates mediating this pain response to MI. They observed several activations within the pain neuromatrix network, correlated to increased pain perception when SCI patients performed MI. Both the ipsilateral premotor cortex and the SMA participated to actual motor processing. When compared to healthy controls, SCI patients elicited greater activation within cM1 attesting that motor output during MI reached the circuitry underlying pain response, due to cerebral reorganizations after the neurological lesion. Therefore, it seems reasonable to assume that motor signals *do* occur during MI, and might act as a triggering stimulus for pain in specific clinical cases of neurological disorders (see above for considerations in amputee patients).

As in the case of stroke, PD, and amputees patients, research investigating MI ability after SCI provides further insight into central processing of motor signals during MI. Using mental chronometry, Decety and Boisson (1990) observed that SCI patients achieved close temporal congruence between MI and PP, whereas brain injured patients failed to do so. These data support the theory that MI accuracy is preserved following SCI because, contrary to what happens after stroke, SCI does not result in cerebral damage. However, SCI elicits consistent reorganization of the sensorimotor cortical maps, with changes in neuronal excitability (Topka et al., 1991; Curt et al., 2002; Dunlop, 2008; Kokotilo et al., 2009) due to deafferentation and deefferentation (Bruehlmeier et al., 1998). As MI is mediated by cerebral substrates overlapping with motor-related regions reorganized consecutively to the lesion, plastic changes after SCI are likely to affect MI ability. Cramer et al. (2005) reported altered MI processing in SCI patients as compared to healthy controls during MI of right foot movements. In this study, task modulation in central activity between MI and PP was absent in SCI patients whereas different brain activation patterns mediated the two tasks in healthy controls. Similarly, Alkadhi et al. (2005) reported that during MI, SCI patients recruited those neural networks that typically mediate both MI and PP in healthy controls. As already mentioned, MI usually results in lower activation intensities than PP in healthy participants, which is typically imputable to inhibitory processes (Porro et al., 1996). Therefore, as early hypothesized by Lacourse et al. (1999), deafferentation and deefferentation following SCI may result in weakened motor command inhibition during MI. SCI patient no longer require to inhibit the mentally rehearsed movement due to the spinal cord lesion, which prevents neural transmissions toward peripheral effectors. This clinical topic is different than that in amputees (see above), in that after SCI patients no longer feel their deafferented and deefferented body regions. Alkadhi et al. (2005) suggested that deafferentation may be a key component for adaptive brain changes regarding inhibition during MI, hence matching the conclusions by Schwoebel et al. (2002). In a recent magnetoencephalographic control-case study (Di Rienzo et al., submitted), a SCI patient presented remarkably similar activations within cM1 during both MI and PP, hence suggesting weakened inhibition of this area during MI. This pattern of activation was associated with disturbed functional network of interrelations between cM1, S1, and SMA. By contrast, a healthy age-matched control participant presented a significant reduction in cM1 activation during MI, with significant inter-relationships in neural activities between cM1 and both SMA and S1. Interestingly, both S1 and SMA are thought to play a key role in motor suppression during MI (Schwoebel et al., 2002; Solodkin et al., 2004; Alkadhi et al., 2005; Kasess et al., 2008). Hotz-Boendermaker et al. (2008) discovered that patients remained nonetheless able to subjectively distinguish MI from attempted PP for actions involving infra-lesional effectors, but the authors mentioned that MI elicited activation of additional brain regions in SCI patients in comparison to controls – presumably to assist motor commands processing. Neuroimaging studies in SCI patients support the theory that central reorganization after SCI results in increased congruence between MI and PP, due to weakened inhibitory processes as a consequence of deafferentation. Accordingly, motor inhibition during MI may have a cortical component involving specific interactions between perirolandic sites and cM1. These results are complementary to those from the TMS approach to understanding inhibition (i.e., intracortical inhibition within M1 and/or incomplete state of CNS facilitation). However, central activity in SCI patients suggests that MI recruits motor programs for overt movements with paralyzed effectors (Sabbah et al., 2002), thereby confirming that these patients preserve the ability to process motor command signals during MI.

A discussed previously, neuroimaging studies support the likelihood of weakened motor inhibition during MI after SCI. However, whether the reductions in sensorimotor activity between MI and PP result in greater transmission of neural signals to the descending pathways during MI remains unknown. Roy et al. (2011) obtained evidence of the downregulation of intracortical inhibition during MI after SCI using paired-pulse TMS. These data support previous findings reporting increased excitability after complete SCI in spared neural pathways (Topka et al., 1991). Reduced intracortical inhibition after SCI is thought to enable unmasking of latent synaptic connections at the cortical level, providing a possible causal mechanism for cerebral plasticity after SCI (Saturno et al., 2008). As MI and PP are assumed to be functionally equivalent, it is plausible that changes in intracortical inhibition of the sensorimotor cortex (allowing the reshaping of actual motor performance) may be reflected during MI, hence confirming some shared neural substrates between these two processes.

Taken together, clinical data from SCI, stroke, amputees, and PD patients converge to suggest that central patterns elicited during MI effectively reflect the internal elaboration of motor commands, although specific clinical impairment provides different and complementary insights to our current knowledge regarding motor inhibition during MI.

## Conclusion

In this paper, we reviewed data that support the emerging hypothesis that both central and peripheral neurophysiological correlates of MI tightly resemble those elicited by actual practice of the same task, even in the absence of overt movement production. Such data also suggest that motor commands are involved during MI. Furthermore, we highlighted evidence that the isomorphism between the representation of imagined and executed actions is preserved, if not strengthened, in specific cases of neurological disorders. Neuroimaging studies clearly support the involvement of both primary and secondary motor-related areas during MI, hence suggesting that neural impulses for motor commands may be elaborated at the cerebral level and addressed, at least partially, from cM1 to the anterior part of the spinal cord via the descending pathways. TMS studies of MI also confirmed this assumption and elucidated our understanding of the neurophysiological processes mediating the involvement of cM1 during MI. Accordingly, corticospinal facilitation during MI might result from changes in excitability at the cortical level. Some authors have also reported that residual EMG activity during MI reflects the features of the motor commands.

Based on these issues, we should now consider the unresolved question of how motor commands are inhibited throughout the motor system to prevent overt execution during MI. We postulate that motor inhibition during MI may not result from a parallel neurophysiological process, concomitant to MI, designed to prevent muscle contractions. First, TMS studies have shown that MI produces opposite effects to those elicited by voluntary relaxation of peripheral effectors regarding corticospinal facilitation (Taniguchi et al., 2008). Secondly, neuroimaging studies have failed to highlight specific neural structures mediating motor inhibition during MI, while TMS data support the idea of increased neuronal excitability and reduced intracortical inhibition within cM1 during MI. A notable finding from neuroimaging research is that secondary motor-related areas like the cerebellum and SMA might play a key role in motor output suppression during MI. Also, impaired sensory feedback integration following deafferentation or brain lesions around the primary somatosensory cortex result in weakened inhibition during MI, thus promoting the role of sensory sites in motor output suppression during MI. Therefore, inhibition during MI may be a functional process resulting from the specific contribution of neural sites usually dedicated to overt motor processing. This theoretical stance might account for the fact that MI activates the motor system in a lesser extent to actual practice.

If we assume that motor inhibition may be intrinsic to the motor command during MI, and that it is potentially mediated by a highly specific interplay between motor-related neural structures, a promising focus of future research may be identified. Specifically, priority for future investigators will be to explore the extent to which cM1 is subjected to active inhibition during MI. Using advanced statistical modeling, several authors have shown that reduced cM1 activation during MI is related to the suppressive influence of other motor-related brain regions, thus suggesting that motor inhibition during MI may also intervene at the early stages of motor planning (Kasess et al., 2008).

**Figure 1:**
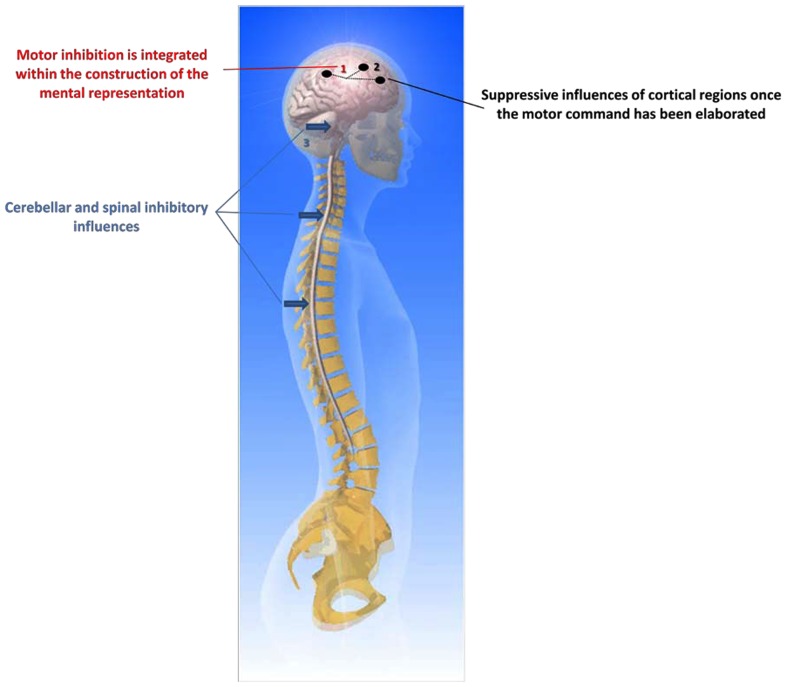
**The three possible routes of motor command inhibition during motor imagery**.

Based on current understandings and literature evidence, we promote a multifactorial explanation of motor inhibition during MI that might involve both cerebral and spinal mechanisms (Table 3). From these findings, we postulate that there are three possible routes of motor command inhibition during MI (Figure 1). One may first hypothesize that motor inhibition is a part of the imagery experience, hence only *subthreshold* motor commands are sent to the effectors to prevent movement execution. A second alternative is the possibility that the inhibitory cerebral regions progressively weaken the motor command during the time course of the MI process, so that only a *residual* activity is sent and can be recorded in the corresponding muscles (for a review on a similar chain of processes during inhibitory motor control in No-Go paradigms, see Band and van Boxtel, 1999). Finally, it is possible that downstream regions including brainstem and spinal influences contribute to motor inhibition at a later stage than in the case of the other two possibilities. There is still, however, one key element requiring further experimental investigation. Specifically, research should establish the degree to which cM1 is inhibited by suppressive neural impulses of cerebral origin during MI. Similarly, it is vital to investigate whether or not neural impulses elicited by MI are blocked by spinal mechanisms triggered by descending input of cerebral origin. Interestingly, inhibition of actual actions in decision making experiments (i.e., Go/No-Go paradigms) or during motor control of complex skills that require real-time adaptation to changing environmental constraints, can happen in the very late stages, and even during overt motor processing. We should also investigate to a greater extent how these inhibitory processes exactly work as these do not operate under a principle of all or nothing. As mentioned by Band and van Boxtel, 1999, p. 190), “*A crucial question is whether it is most important to define the locus of* [motor] *inhibition by the source of inhibitory activity (agent), by the process at which inhibition was exerted (site), or by the location where reduction of response activity can be recorded (manifestation)*.” An important aspect is that imagined movement may be accompanied by reduced motor activity at a level which the subject is unaware of, such as when voluntarily performing movements simultaneously with its own action representation for improving MI vividness. Therefore, as the functional equivalence between imagined and executed actions has mainly been considered at the scope of neurophysiological correlates in activation, it might also be observable through the neural processes mediating motor output suppression during these two behaviors.

**Table 3:** **Studies addressing the question of the motor inhibition during motor imagery**.

Authors	Type of study	Method	Participants	Potential inhibitory regions
Alkadhi et al. (2005)	Motor imagery of foot movement	fMRI	Healthy (*n* = 8) Patients (*n* = 8)	Motor command suppression but no clear inhibitory regions
Bonnet et al. (1997)	Motor imagery of a foot pressure on a pedal	Reflex stimulation	Healthy (*n* = 20)	Inhibitory spinal influences
Di Rienzo et al. (submitted)	Case study with a C6–C7 quadriplegic patient	MEG	Patients (*n* = 1)	Primary sensory area and supplementary motor area
Jeannerod (2001, 2006)	Review papers	–	–	prefrontal cortical areas and/or brainstem and spinal influences
Kasess et al. (2008)	Motor imagery of finger movements	fMRI	Healthy (*n* = 8)	Supplementary motor area
Lotze et al. (1999b)	Motor imagery of hand movements	fMRI	Healthy (*n* = 10)	Posterior cerebellum
Schwoebel et al. (2002)	Case study with a patient suffering from bilateral parietal lesions	Psychophysic experiment	Patients (*n* = 1)	Fronto-parietal network
Solodkin et al. (2004)	Motor imagery of a finger-to-thumb opposition task	fMRI	Healthy (*n* = 18)	Superior parietal lobule and supplementary motor area
Deiber et al. (1998)	Motor imagery of finger movements	TEP	Healthy (*n* = 10)	Inferior frontal cortex
Lebon et al. (2012)	Motor imagery and mental rotation of a pinching movement	TMS	Healthy (*n* = 11)	Inferior parietal lobule

## Conflict of Interest Statement

The authors declare that the research was conducted in the absence of any commercial or financial relationships that could be construed as a potential conflict of interest.

## References

[B1] Abbruzzese G. Assini A. Buccolieri A. Marchese R. Trompetto C. (1999). Changes of intracortical inhibition during motor imagery in human subjects. Neurosci. Lett. 263, 113–116 10.1016/S0304-3940(99)00120-2 10213148

[B2] Alkadhi H. Brugger P. Boendermaker S. H. Crelier G. Curt A. Hepp-Reymond M. C. Kollias S. S. (2005). What disconnection tells about motor imagery: evidence from paraplegic patients. Cereb. Cortex 15, 131–140 10.1093/cercor/bhh116 15238440

[B3] Aron A. R. Durston S. Eagle D. M. Logan G. D. Stinear C. M. Stuphorn V. (2007). Converging evidence for a fronto-basal-ganglia network for inhibitory control of action and cognition. J. Neurosci. 27, 11860–11864 10.1523/JNEUROSCI.0519-07.2007 17978025 PMC6673355

[B4] Bakker F. C. Boschker M. S. J. Chung T. (1996). Changes in muscular activity while imagining weight lifting using stimulus or response propositions. J. Sport Exerc. Psychol. 18, 313–324

[B5] Band G. P. H. van Boxtel G. J. M. (1999). Inhibitory motor control in stop paradigms: review and reinterpretation of neural mechanisms. Acta Psychol. (Amst.) 101, 179–211 10.1016/S0001-6918(99)00005-0 10344185

[B6] Battaglia F. Quartarone A. Ghilardi M. F. Dattola R. Bagnato S. Rizzo V. Morgante L. Girlanda P. (2006). Unilateral cerebellar stroke disrupts movement preparation and motor imagery. Clin. Neurophysiol. 117, 1009–1016 10.1016/j.clinph.2006.01.008 16516543

[B7] Bird E. I. (1984). EMG quantification of mental rehearsal. Percept. Mot. Skills 59, 899–906 10.2466/pms.1984.59.3.899

[B8] Bonnet M. Decety J. Jeannerod M. Requin J. (1997). Mental simulation of an action modulates the excitability of spinal reflex pathways in man. Brain Res. Cogn. Brain Res. 5, 221–228 10.1016/S0926-6410(96)00072-9 9088558

[B9] Boschker M. S. J. (2001). Action-Based Imagery: On the Nature of Mentally Imagined Motor Actions. Amsterdam: University of Amsterdam

[B10] Boucher L. Palmeri T. J. Logan G. D. Schall J. D. (2007). Inhibitory control in mind and brain: an interactive race model of countermanding saccades. Psychol. Rev. 114, 376–397 10.1037/0033-295X.114.2.376 17500631

[B11] Braun S. M. Beurskens A. J. Borm P. J. Schack T. Wade D. T. (2006). The effects of mental practice in stroke rehabilitation: a systematic review. Arch. Phys. Med. Rehabil. 87, 842–852 10.1016/j.apmr.2006.02.034 16731221

[B12] Braun S. Beurskens A. Kleynen M. Schols J. Wade D. (2011). Rehabilitation with mental practice has similar effects on mobility as rehabilitation with relaxation in people with Parkinson’s disease: a multicentre randomised trial. J. Physiother. 57, 27–34 10.1016/S1836-9553(11)70004-2 21402327

[B13] Bruehlmeier M. Dietz V. Leenders K. L. Roelcke U. Missimer J. Curt A. (1998). How does the human brain deal with a spinal cord injury? Eur. J. Neurosci. 10, 3918–3922 10.1046/j.1460-9568.1998.00454.x 9875370

[B14] Brunia C. H. M. (2003). “How is stopping realized?” in Errors, Conflicts, and The Brain. Current Opinions on Performance Monitoring, eds Ullsperger M. Falkenstein M. (Liepzig: MPI of Cognitive Neuroscience), 96–103

[B15] Callow N. Waters A. (2005). The effect of kinesthetic imagery on the sport confidence of flat-race horse jockeys. Psychol. Sport. Exerc. 6, 443–459 10.1016/j.psychsport.2004.08.001

[B16] Carpenter W. B. (1894). Principles of Mental Physiology. Appleton: D. Appleton

[B17] Chan B. L. Witt R. Charrow A. P. Magee A. Howard R. Pasquina P. F. Heilman K. M. Tsao J. W. (2007). Mirror therapy for phantom limb pain. N. Engl. J. Med. 357, 2206–2207 10.1056/NEJMicm063551 18032777

[B18] Clark S. Tremblay F. Ste-Marie D. (2004). Differential modulation of corticospinal excitability during observation, mental imagery and imitation of hand actions. Neuropsychologia 42, 105–112 10.1016/S0028-3932(03)00144-1 14615080

[B19] Cohen R. G. Chao A. Nutt J. G. Horak F. B. (2011). Freezing of gait is associated with a mismatch between motor imagery and motor execution in narrow doorways, not with failure to judge doorway passability. Neuropsychologia 49, 3981–3988 10.1016/j.neuropsychologia.2011.10.014 22027173 PMC3260879

[B20] Collet C. Guillot A. (2010). “Autonomic nervous system activities during imagined movements,” in The Neurophysiological Foundations of Mental and Motor Imagery, eds Guillot A. Collet C. (New York: Oxford University Press), 95–108

[B21] Cramer S. C. Lastra L. Lacourse M. G. Cohen M. J. (2005). Brain motor system function after chronic, complete spinal cord injury. Brain 128, 2941–2950 10.1093/brain/awh648 16246866

[B22] Cunnington R. Egan G. F. O’Sullivan J. D. Hughes A. J. Bradshaw J. L. Colebatch J. G. (2001). Motor imagery in Parkinson’s disease: a PET study. Mov. Disord. 16, 849–857 10.1002/mds.1181 11746614

[B23] Cunnington R. Iansek R. Johnson K. A. Bradshaw J. L. (1997). Movement-related potentials in Parkinson’s disease. Motor imagery and movement preparation. Brain 120(Pt 8), 1339–1353 10.1093/brain/120.8.1339 9278627

[B24] Curt A. Alkadhi H. Crelier G. R. Boendermaker S. H. Hepp-Reymond M. C. Kollias S. S. (2002). Changes of non-affected upper limb cortical representation in paraplegic patients as assessed by fMRI. Brain 125, 2567–2578 10.1093/brain/awf250 12390981

[B25] Daprati E. Nico D. Duval S. Lacquaniti F. (2010). Different motor imagery modes following brain damage. Cortex 46, 1016–1030 10.1016/j.cortex.2009.08.002 19726037

[B26] de Jong R. Coles M. G. Logan G. D. Gratton G. (1990). In search of the point of no return: the control of response processes. J. Exp. Psychol. Hum. Percept. Perform. 16, 164–182 10.1037/0096-1523.16.1.164 2137517

[B27] de Vries S. Mulder T. (2007). Motor imagery and stroke rehabilitation: a critical discussion. J. Rehabil. Med. 39, 5–13 10.2340/16501977-0020 17225031

[B28] Decety J. Boisson D. (1990). Effect of brain and spinal cord injuries on motor imagery. Eur. Arch. Psychiatry Clin. Neurosci. 240, 39–43 10.1007/BF02190091 2147901

[B29] Decety J. Grezes J. (1999). Neural mechanisms subserving the perception of human actions. Trends Cogn. Sci. (Regul. Ed.) 3, 172–178 10.1016/S1364-6613(99)01312-1 10322473

[B30] Decety J. Jeannerod M. Durozard D. Baverel G. (1993). Central activation of autonomic effectors during mental simulation of motor actions in man. J. Physiol. (Lond.) 461, 549–563 8102402 10.1113/jphysiol.1993.sp019528 PMC1175272

[B31] Dechent P. Merboldt K. D. Frahm J. (2004). Is the human primary motor cortex involved in motor imagery? Brain Res. Cogn. Brain Res. 19, 138–144 10.1016/j.cogbrainres.2003.11.012 15019710

[B32] Deiber M. P. Ibanez V. Honda M. Sadato N. Raman R. Hallett M. (1998). Cerebral processes related to visuomotor imagery and generation of simple finger movements studied with positron emission tomography. Neuroimage 7, 73–85 10.1006/nimg.1997.0314 9571132

[B33] Demougeot L. Papaxanthis C. (2011). Muscle fatigue affects mental simulation of action. J. Neurosci. 31, 10712–10720 10.1523/JNEUROSCI.6032-10.2011 21775614 PMC6622640

[B34] Dettmers C. Benz M. Liepert J. Rockstroh B. (2012). Motor imagery in stroke patients, or plegic patients with spinal cord or peripheral diseases. Acta Neurol. Scand. (in press). 10.1111/j.1600-0404.2012.01680.x 22587653

[B35] Dickstein R. Deutsch J. E. (2007). Motor imagery in physical therapist practice. Phys. Ther. 87, 942–953 10.2522/ptj.20060331 17472948

[B36] Dickstein R. Gazit-Grunwald M. Plax M. Dunsky A. Marcovitz E. (2005). EMG activity in selected target muscles during imagery rising on tiptoes in healthy adults and poststroke hemiparetic patients. J. Mot. Behav. 37, 475–483 10.3200/JMBR.37.6.475-483 16280318

[B37] Diers M. Christmann C. Koeppe C. Ruf M. Flor H. (2010). Mirrored, imagined and executed movements differentially activate sensorimotor cortex in amputees with and without phantom limb pain. Pain 149, 296–304 10.1016/j.pain.2010.02.020 20359825

[B38] Dominey P. Decety J. Broussolle E. Chazot G. Jeannerod M. (1995). Motor imagery of a lateralized sequential task is asymmetrically slowed in hemi-Parkinson’s patients. Neuropsychologia 33, 727–741 10.1016/0028-3932(95)00008-Q 7675164

[B39] Driskell J. E. Cooper C. Moran A. (1994). Does mental practice enhance performance? J. Appl. Psychol. 79, 481–492 10.1037/0021-9010.79.4.481

[B40] Duclos Y. Schmied A. Burle B. Burnet H. Rossi-Durand C. (2008). Anticipatory changes in human motoneuron discharge patterns during motor preparation. J. Physiol. (Lond.) 586, 1017–1028 10.1113/jphysiol.2007.145318 18079160 PMC2375638

[B41] Dunlop S. A. (2008). Activity-dependent plasticity: implications for recovery after spinal cord injury. Trends Neurosci. 31, 410–418 10.1016/j.tins.2008.05.004 18602172

[B42] Ehde D. M. Czerniecki J. M. Smith D. G. Campbell K. M. Edwards W. T. Jensen M. P. Robinson L. R. (2000). Chronic phantom sensations, phantom pain, residual limb pain, and other regional pain after lower limb amputation. Arch. Phys. Med. Rehabil. 81, 1039–1044 10.1053/apmr.2000.7583 10943752

[B43] Ehrsson H. H. Geyer S. Naito E. (2003). Imagery of voluntary movement of fingers, toes, and tongue activates corresponding body-part-specific motor representations. J. Neurophysiol. 90, 3304–3316 10.1152/jn.01113.2002 14615433

[B44] Ersland L. Rosen G. Lundervold A. Smievoll A. I. Tillung T. Sundberg H. Hugdahl K. (1996). Phantom limb imaginary fingertapping causes primary motor cortex activation: an fMRI study. Neuroreport 8, 207–210 10.1097/00001756-199612200-00042 9051782

[B45] Facchini S. Muellbacher W. Battaglia F. Boroojerdi B. Hallett M. (2002). Focal enhancement of motor cortex excitability during motor imagery: a transcranial magnetic stimulation study. Acta Neurol. Scand. 105, 146–151 10.1034/j.1600-0404.2002.1o004.x 11886355

[B46] Fadiga L. Buccino G. Craighero L. Fogassi L. Gallese V. Pavesi G. (1999). Corticospinal excitability is specifically modulated by motor imagery: a magnetic stimulation study. Neuropsychologia 37, 147–158 10.1016/S0028-3932(98)00089-X 10080372

[B47] Flor H. Elbert T. Knecht S. Wienbruch C. Pantev C. Birbaumer N. Larbig W. Taub E. (1995). Phantom-limb pain as a perceptual correlate of cortical reorganization following arm amputation. Nature 375, 482–484 10.1038/375482a0 7777055

[B48] Fourkas A. D. Avenanti A. Urgesi C. Aglioti S. M. (2006). Corticospinal facilitation during first and third person imagery. Exp. Brain Res. 168, 143–151 10.1007/s00221-005-0076-0 16044298

[B49] Gandevia S. C. (1999). Mind, muscles, and motoneurones. J. Sci. Med. Sport. 2, 167–180 10.1016/S1440-2440(99)80171-6 10668756

[B50] Gandevia S. C. Wilson L. R. Inglis J. T. Burke D. (1997). Mental rehearsal of motor tasks recruits alpha-motoneurones but fails to recruit human fusimotor neurones selectively. J. Physiol. (Lond.) 505(Pt 1), 259–266 10.1111/j.1469-7793.1997.259bc.x 9409487 PMC1160109

[B51] Gao Q. Duan X. Chen H. (2011). Evaluation of effective connectivity of motor areas during motor imagery and execution using conditional Granger causality. Neuroimage 54, 1280–1288 10.1016/j.neuroimage.2010.08.071 20828626

[B52] Garavan H. Ross T. J. Murphy K. Roche R. A. Stein E. A. (2002). Dissociable executive functions in the dynamic control of behavior: inhibition, error detection, and correction. Neuroimage 17, 1820–1829 10.1006/nimg.2002.1326 12498755

[B53] Gentili R. Papaxanthis C. Pozzo T. (2006). Improvement and generalization of arm motor performance through imagery practice. Neuroscience 137, 761–772 10.1016/j.neuroscience.2005.10.013 16338093

[B54] Gerardin E. Sirigu A. Lehericy S. Poline J. B. Gaymard B. Marsault C. Agid Y. Le Bihan D. (2000). Partially overlapping neural networks for real and imagined hand movements. Cereb. Cortex 10, 1093–1104 10.1093/cercor/10.11.1093 11053230

[B55] Gonzalez B. Rodriguez M. Ramirez C. Sabate M. (2005). Disturbance of motor imagery after cerebellar stroke. Behav. Neurosci. 119, 622–626 10.1037/0735-7044.119.2.622 15839808

[B56] Gray J. A. (1990). Brain systems that mediate both emotion and cognition. Cogn. Emot. 4, 269–288 10.1080/02699939008410799

[B57] Grezes J. Decety J. (2001). Functional anatomy of execution, mental simulation, observation, and verb generation of actions: a meta-analysis. Hum. Brain Mapp. 12, 1–19 10.1002/1097-0193(200101)12:1<1::AID-HBM10>3.0.CO;2-V 11198101 PMC6872039

[B58] Gueugneau N. Crognier L. Papaxanthis C. (2008). The influence of eye movements on the temporal features of executed and imagined arm movements. Brain Res. 1187, 95–102 10.1016/j.brainres.2007.10.042 18035337

[B59] Guillot A. Collet C. (2008). Construction of the motor imagery integrative model in sport: a review and theoretical investigation of motor imagery use. Int. Rev. Sport Exerc. Psychol. 1, 31–44 10.1080/17509840701823139

[B60] Guillot A. Collet C. Nguyen V. A. Malouin F. Richards C. Doyon J. (2008). Functional neuroanatomical networks associated with expertise in motor imagery. Neuroimage 41, 1471–1483 10.1016/j.neuroimage.2008.03.042 18479943

[B61] Guillot A. Collet C. Nguyen V. A. Malouin F. Richards C. Doyon J. (2009). Brain activity during visual versus kinesthetic imagery: an fMRI study. Hum. Brain Mapp. 30, 2157–2172 10.1002/hbm.20658 18819106 PMC6870928

[B62] Guillot A. Di Rienzo F. Collet C. (2012). “The neurofunctional architecture of motor imagery,” in Functional Magnetic Resonance Imaging/Book 1, eds Papageorgiou T. D. Christopoulos G. Smirnakis S. (InTech) (in press).

[B63] Guillot A. Lebon F. Collet C. (2010). “Electromyographic activity during motor imagery,” in The Neurophysiological Foundations of Mental and Motor Imagery, eds Guillot A. Collet C. (New York: Oxford University Press), 83–93

[B64] Guillot A. Lebon F. Rouffet D. Champely S. Doyon J. Collet C. (2007). Muscular responses during motor imagery as a function of muscle contraction types. Int. J. Psychophysiol. 66, 18–27 10.1016/j.ijpsycho.2007.05.009 17590469

[B65] Gustin S. M. Wrigley P. J. Gandevia S. C. Middleton J. W. Henderson L. A. Siddall P. J. (2008). Movement imagery increases pain in people with neuropathic pain following complete thoracic spinal cord injury. Pain 137, 237–244 10.1016/j.pain.2007.08.032 17942228

[B66] Gustin S. M. Wrigley P. J. Henderson L. A. Siddall P. J. (2010). Brain circuitry underlying pain in response to imagined movement in people with spinal cord injury. Pain 148, 438–445 10.1016/j.pain.2009.12.001 20092946

[B67] Hale B. D. (1982). The effects of internal and external imagery on muscular and ocular concomitants. J. Sport Psychol. 4, 379–387

[B68] Hale B. S. Raglin J. S. Koceja D. M. (2003). Effect of mental imagery of a motor task on the Hoffmann reflex. Behav. Brain Res. 142, 81–87 10.1016/S0166-4328(02)00397-2 12798268

[B69] Hanakawa T. Dimyan M. A. Hallett M. (2008). Motor planning, imagery, and execution in the distributed motor network: a time-course study with functional MRI. Cereb. Cortex 18, 2775–2788 10.1093/cercor/bhn036 18359777 PMC2583155

[B70] Hanakawa T. Immisch I. Toma K. Dimyan M. A. Van Gelderen P. Hallett M. (2003). Functional properties of brain areas associated with motor execution and imagery. J. Neurophysiol. 89, 989–1002 10.1152/jn.00132.2002 12574475

[B71] Harris A. J. (1999). Cortical origin of pathological pain. Lancet 354, 1464–1466 10.1016/S0140-6736(99)05003-5 10543687

[B72] Harris D. V. Robinson W. J. (1986). The effects of skill level on EMG activity during internal and external imagery. J. Sport Psychol. 8, 105–111

[B73] Hashimoto R. Rothwell J. C. (1999). Dynamic changes in corticospinal excitability during motor imagery. Exp. Brain Res. 125, 75–81 10.1007/s002210050693 10100979

[B74] Helmich R. C. Bloem B. R. Toni I. (2012). Motor imagery evokes increased somatosensory activity in parkinson’s disease patients with tremor. Hum. Brain Mapp. 33, 1763–1779 10.1002/hbm.21318 21674693 PMC6869863

[B75] Helmich R. C. De Lange F. P. Bloem B. R. Toni I. (2007). Cerebral compensation during motor imagery in Parkinson’s disease. Neuropsychologia 45, 2201–2215 10.1016/j.neuropsychologia.2007.02.024 17448507

[B76] Heremans E. Feys P. Nieuwboer A. Vercruysse S. Vandenberghe W. Sharma N. Helsen W. (2011). Motor imagery ability in patients with early- and mid-stage Parkinson disease. Neurorehabil. Neural Repair 25, 168–177 10.1177/1545968310370750 21239707

[B77] Heremans E. Helsen W. F. Feys P. (2008). The eyes as a mirror of our thoughts: quantification of motor imagery of goal-directed movements through eye movement registration. Behav. Brain Res. 187, 351–360 10.1016/j.bbr.2007.09.028 17977607

[B78] Heremans E. Nieuwboer A. Feys P. Vercruysse S. Vandenberghe W. Sharma N. Helsen W. F. (2012). External cueing improves motor imagery quality in patients with Parkinson disease. Neurorehabil. Neural Repair 26, 27–35 10.1177/1545968311411055 21778409

[B79] Holmes P. S. Collins D. J. (2001). The PETTLEP approach to motor imagery: a functional equivalence model for sport psychologists. J. Appl. Sport Psychol. 13, 60–83 10.1080/10413200109339004

[B80] Hotz-Boendermaker S. Funk M. Summers P. Brugger P. Hepp-Reymond M. C. Curt A. Kollias S. S. (2008). Preservation of motor programs in paraplegics as demonstrated by attempted and imagined foot movements. Neuroimage 39, 383–394 10.1016/j.neuroimage.2007.07.065 17919932

[B81] Jackson P. L. Lafleur M. F. Malouin F. Richards C. L. Doyon J. (2003). Functional cerebral reorganization following motor sequence learning through mental practice with motor imagery. Neuroimage 20, 1171–1180 10.1016/S1053-8119(03)00369-0 14568486

[B82] Jacobson E. (1930). Electrical measurements of neuromuscular states during mental activities. Am. J. Physiol. 91, 567–608

[B83] Jacobson E. (1932). Electrophysiology of mental activities. Am. J. Psychol. 44, 677–694 10.2307/1414531

[B84] Jeannerod M. (1994). The representing brain: neural correlates of motor intention and imagery. Behav. Brain Sci. 17, 187–202 10.1017/S0140525X0003435X

[B85] Jeannerod M. (2001). Neural simulation of action: a unifying mechanism for motor cognition. Neuroimage 14, S103–S109 10.1006/nimg.2001.0832 11373140

[B86] Jeannerod M. (2006). Motor Cognition. New York: Oxford University Press

[B87] Jeannerod M. Frak V. (1999). Mental imaging of motor activity in humans. Curr. Opin. Neurobiol. 9, 735–739 10.1016/S0959-4388(99)00038-0 10607647

[B88] Johnson S. H. Sprehn G. Saykin A. J. (2002). Intact motor imagery in chronic upper limb hemiplegics: evidence for activity-independent action representations. J. Cogn. Neurosci. 14, 841–852 10.1162/089892902760191072 12191452

[B89] Jowdy D. P. Harris D. V. (1990). Muscular responses during mental imagery as a function of motor skill level. J. Sport Exerc. Psychol. 12, 191–201

[B90] Kagerer F. A. Bracha V. Wunderlich D. A. Stelmach G. E. Bloedel J. R. (1998). Ataxia reflected in the simulated movements of patients with cerebellar lesions. Exp. Brain Res. 121, 125–134 10.1007/s002210050444 9696381

[B91] Karl A. Birbaumer N. Lutzenberger W. Cohen L. G. Flor H. (2001). Reorganization of motor and somatosensory cortex in upper extremity amputees with phantom limb pain. J. Neurosci. 21, 3609–3618 11331390 10.1523/JNEUROSCI.21-10-03609.2001 PMC6762494

[B92] Kasai T. Kawai S. Kawanishi M. Yahagi S. (1997). Evidence for facilitation of motor evoked potentials (MEPs) induced by motor imagery. Brain Res. 744, 147–150 10.1016/S0006-8993(96)01101-8 9030424

[B93] Kasess C. H. Windischberger C. Cunnington R. Lanzenberger R. Pezawas L. Moser E. (2008). The suppressive influence of SMA on M1 in motor imagery revealed by fMRI and dynamic causal modeling. Neuroimage 40, 828–837 10.1016/j.neuroimage.2007.11.040 18234512

[B94] Kimberley T. J. Khandekar G. Skraba L. L. Spencer J. A. Van Gorp E. A. Walker S. R. (2006). Neural substrates for motor imagery in severe hemiparesis. Neurorehabil. Neural Repair 20, 268–277 10.1177/1545968306286958 16679504

[B95] Kleber B. Birbaumer N. Veit R. Trevorrow T. Lotze M. (2007). Overt and imagiend singing of an Italian aria. Neuroimage 36, 1238–1246 10.1016/j.neuroimage.2007.02.053 17478107

[B96] Knecht S. Henningsen H. Elbert T. Flor H. Hohling C. Pantev C. Taub E. (1996). Reorganizational and perceptional changes after amputation. Brain 119(Pt 4), 1213–1219 10.1093/brain/119.4.1213 8813284

[B97] Kokotilo K. J. Eng J. J. Curt A. (2009). Reorganization and preservation of motor control of the brain in spinal cord injury: a systematic review. J. Neurotrauma 26, 2113–2126 10.1089/neu.2008.0688 19604097 PMC3167869

[B98] Kosslyn S. M. (2010). “Multimotal images in the brain,” in The Neurophysiological Foundations of Mental and Motor Imagery, eds Guillot A. Collet C. (New York, NY: Oxford University Press), 3–16

[B99] Kosslyn S. M. Ganis G. Thompson W. L. (2001). Neural foundations of imagery. Nat. Rev. Neurosci. 2, 635–642 10.1038/35085102 11533731

[B100] Kuhn A. A. Doyle L. Pogosyan A. Yarrow K. Kupsch A. Schneider G. H. Hariz M. I. Trottenberg T. Brown P. (2006). Modulation of beta oscillations in the subthalamic area during motor imagery in Parkinson’s disease. Brain 129, 695–706 10.1093/brain/awh715 16364953

[B101] Kumru H. Soto O. Casanova J. Valls-Sole J. (2008). Motor cortex excitability changes during imagery of simple reaction time. Exp. Brain Res. 189, 373–378 10.1007/s00221-008-1433-6 18512049

[B102] Lacourse M. G. Cohen M. J. Lawrence K. E. Romero D. H. (1999). Cortical potentials during imagined movements in individuals with chronic spinal cord injuries. Behav. Brain Res. 104, 73–88 10.1016/S0166-4328(99)00052-2 11125744

[B103] Lafleur M. F. Jackson P. L. Malouin F. Richards C. L. Evans A. C. Doyon J. (2002). Motor learning produces parallel dynamic functional changes during the execution and imagination of sequential foot movements. Neuroimage 16, 142–157 10.1006/nimg.2001.1048 11969325

[B104] Lebon F. Byblow W. D. Collet C. Guillot A. Stinear C. M. (2012). The modulation of motor cortex excitability during motor imagery depends on imagery quality. Eur. J. Neurosci. 35, 323–331 10.1111/j.1460-9568.2011.07938.x 22172012

[B105] Lebon F. Rouffet D. Collet C. Guillot A. (2008). Modulation of EMG power spectrum frequency during motor imagery. Neurosci. Lett. 435, 181–185 10.1016/j.neulet.2008.02.033 18343579

[B106] Lenartowicz A. Verbruggen F. Logan G. D. Poldrack R. A. (2011). Inhibition-related activation in the right inferior frontal gyrus in the absence of inhibitory cues. J. Cogn. Neurosci. 23, 3388–3399 10.1162/jocn_a_00031 21452946

[B107] Leonard G. Tremblay F. (2007). Corticomotor facilitation associated with observation, imagery and imitation of hand actions: a comparative study in young and old adults. Exp. Brain Res. 177, 167–175 10.1007/s00221-006-0657-6 16947064

[B108] Li S. Kamper D. G. Stevens J. A. Rymer W. Z. (2004a). The effect of motor imagery on spinal segmental excitability. J. Neurosci. 24, 9674–9680 10.1523/JNEUROSCI.1370-04.2004 15509755 PMC6730147

[B109] Li S. Latash M. L. Zatsiorsky V. M. (2004b). Effects of motor imagery on finger force responses to transcranial magnetic stimulation. Brain Res. Cogn. Brain Res. 20, 273–280 10.1016/j.cogbrainres.2004.03.003 15183398

[B110] Liang N. Ni Z. Takahashi M. Murakami T. Yahagi S. Funase K. Kato T. Kasai T. (2007). Effects of motor imagery are dependent on motor strategies. Neuroreport 18, 1241–1245 10.1097/WNR.0b013e3282202707 17632275

[B111] Liepert J. Greiner J. Nedelko V. Dettmers C. (2012). Reduced upper limb sensation impairs mental chronometry for motor imagery after stroke: clinical and electrophysiological findings. Neurorehabil. Neural Repair 26, 470–478 10.1177/1545968311425924 22247502

[B112] Lim V. K. Polych M. A. Hollander A. Byblow W. D. Kirk I. J. Hamm J. P. (2006). Kinesthetic but not visual imagery assists in normalizing the CNV in Parkinson’s disease. Clin. Neurophysiol. 117, 2308–2314 10.1016/j.clinph.2006.06.713 16890482

[B113] Livesay J. R. Samaras M. R. (1998). Covert neuromuscular activity of the dominant forearm during visualization of a motor task. Percept. Mot. Skills 86, 371–374 10.2466/pms.1998.86.2.371 10049098

[B114] Logan G. D. (1983). On the ability to inhibit simple thoughts and actions: I. Stop signal studies of decision and memory. J. Exp. Psychol. Learn. Mem. Cogn. 9, 585–606 10.1037/0278-7393.9.4.585

[B115] Logan G. D. (1985). On the ability to inhibit simple thoughts and actions: II. Stop-signal studies of repetition priming. J. Exp. Psychol. Learn. Mem. Cogn. 11, 675–691 10.1037/0278-7393.11.1-4.675

[B116] Logan G. D. Cowan W. B. (1984). On the ability to inhibit thought and action: a theory of an act of control. Psychol. Rev. 91, 295–327 10.1037/0033-295X.91.3.295 24490789

[B117] Lorey B. Bischoff M. Pilgramm S. Stark R. Munzert J. Zentgraf K. (2009). The embodied nature of motor imagery: the influence of posture and perspective. Exp. Brain Res. 194, 233–243 10.1007/s00221-008-1693-1 19139856

[B118] Lotze M. Flor H. Grodd W. Larbig W. Birbaumer N. (2001). Phantom movements and pain. An fMRI study in upper limb amputees. Brain 124, 2268–2277 10.1093/brain/124.11.2268 11673327

[B119] Lotze M. Grodd W. Birbaumer N. Erb M. Huse E. Flor H. (1999a). Does use of a myoelectric prosthesis prevent cortical reorganization and phantom limb pain? Nat. Neurosci. 2, 501–502 10.1038/9145 10448212

[B120] Lotze M. Montoya P. Erb M. Hulsmann E. Flor H. Klose U. Birbaumer N. Grodd W. (1999b). Activation of cortical and cerebellar motor areas during executed and imagined hand movements: an fMRI study. J. Cogn. Neurosci. 11, 491–501 10.1162/089892999563553 10511638

[B121] Lotze M. Halsband U. (2006). Motor imagery. J. Physiol. Paris 99, 386–395 10.1016/j.jphysparis.2006.03.012 16716573

[B122] Lotze M. Scheler G. Tan H. R. Braun C. Birbaumer N. (2003). The musician’s brain: functional imaging of amateurs and professionals during performance and imagery. Neuroimage 20, 1817–1829 10.1016/j.neuroimage.2003.07.018 14642491

[B123] Lotze M. Zentgraf K. (2010). “Contribution of the primary motor cortex to motor imagery,” in The Neurophysiological Foundations of Mental and Motor Imagery, eds Guillot A. Collet C. (New York: Oxford University Press), 31–45

[B124] Louis M. Collet C. Champely S. Guillot A. (2012). Differences in motor imagery time when predicting task duration in skiers and equestrian riders. Res. Q. Exerc. Sport 83, 86–93 22428415 10.1080/02701367.2012.10599828

[B125] Luppino G. Matelli M. Camarda R. Rizzolatti G. (1994). Corticospinal projections from mesial frontal and cingulate areas in the monkey. Neuroreport 5, 2545–2548 10.1097/00001756-199412000-00035 7696600

[B126] Lutz R. Linder D. E. (2001). Does electromyographic activity during motor imagery predict performance? A test of bioinformational theory and functional equivalences. J. Sport Exerc. Psychol. 23, s63

[B127] MacIver K. Lloyd D. M. Kelly S. Roberts N. Nurmikko T. (2008). Phantom limb pain, cortical reorganization and the therapeutic effect of mental imagery. Brain 131, 2181–2191 10.1093/brain/awn124 18567624 PMC2494616

[B128] Macuga K. L. Frey S. H. (2012). Neural representations involved in observed, imagined, and imitated actions are dissociable and hierarchically organized. Neuroimage 59, 2798–2807 10.1016/j.neuroimage.2011.09.083 22005592 PMC3254825

[B129] Malouin F. Richards C. L. Desrosiers J. Doyon J. (2004). Bilateral slowing of mentally simulated actions after stroke. Neuroreport 15, 1349–1353 10.1097/01.wnr.0000127465.94899.72 15167564

[B130] Malouin F. Richards C. L. Durand A. Doyon J. (2008). Clinical assessment of motor imagery after stroke. Neurorehabil. Neural Repair 22, 330–340 18326057 10.1177/1545968307313499

[B131] Mannix L. K. Chandurkar R. S. Rybicki L. A. Tusek D. L. Solomon G. D. (1999). Effect of guided imagery on quality of life for patients with chronic tension-type headache. Headache 39, 326–334 10.1046/j.1526-4610.1999.3905326.x 11279912

[B132] Marconi B. Pecchioli C. Koch G. Caltagirone C. (2007). Functional overlap between hand and forearm motor cortical representations during motor cognitive tasks. Clin. Neurophysiol. 118, 1767–1775 10.1016/j.clinph.2007.08.021 17576095

[B133] Mayer A. Kudar K. Bretz K. Tihanyi J. (2008). Body schema and body awareness of amputees. Prosthet. Orthot. Int. 32, 363–382 10.1080/03093640802024971 18677671

[B134] Mercier C. Sirigu A. (2009). Training with virtual visual feedback to alleviate phantom limb pain. Neurorehabil. Neural Repair 23, 587–594 10.1177/1545968308328717 19171946

[B135] Michelon P. Vettel J. M. Zacks J. M. (2006). Lateral somatotopic organization during imagined and prepared movements. J. Neurophysiol. 95, 811–822 10.1152/jn.00488.2005 16207787

[B136] Milton J. Solodkin A. Hlustik P. Small S. L. (2007). The mind of expert motor performance is cool and focused. Neuroimage 35, 804–813 10.1016/j.neuroimage.2007.01.003 17317223

[B137] Moran A. Guillot A. MacIntyre T. Collet C. (2012). Re-imagining motor imagery: building bridges between cognitive neuroscience and sport psychology. Br. J. Psychol. 103, 224–247 10.1111/j.2044-8295.2011.02068.x 22506748

[B138] Morecraft R. J. Herrick J. L. Stilwell-Morecraft K. S. Louie J. L. Schroeder C. M. Ottenbacher J. G. Schoolfield M. W. (2002). Localization of arm representation in the corona radiata and internal capsule in the non-human primate. Brain 125, 176–198 10.1093/brain/awf011 11834603

[B139] Mulder T. De Vries S. Zijlstra S. (2005). Observation, imagination and execution of an effortful movement: more evidence for a central explanation of motor imagery. Exp. Brain Res. 163, 344–351 10.1007/s00221-004-2179-4 15654585

[B140] Mulder T. Zijlstra S. Zijlstra W. Hochstenbach J. (2004). The role of motor imagery in learning a totally novel movement. Exp. Brain Res. 154, 211–217 10.1007/s00221-003-1647-6 14508635

[B141] Munzert J. Lorey B. Zentgraf K. (2009). Cognitive motor processes: the role of motor imagery in the study of motor representations. Brain Res. Rev. 60, 306–326 10.1016/j.brainresrev.2008.12.024 19167426

[B142] Murphy S. Nordin S. M. Cumming J. (2008). “Imagery in sport, exercise and dance,” in Advances in Sport Psychology, ed. Horn T. (Champaign, IL: Human Kinetics), 306–315

[B143] Naito E. Kochiyama T. Kidata R. Nakamura S. Matsumura M. Yonekura Y. Sadato N. (2002). Internally simulated movement sensations during motor imagery activate cortical motor areas and the cerebellum. J. Neurosci. 22, 3683–3691 11978844 10.1523/JNEUROSCI.22-09-03683.2002 PMC6758350

[B144] Nico D. Daprati E. Rigal F. Parsons L. Sirigu A. (2004). Left and right hand recognition in upper limb amputees. Brain 127, 120–132 10.1093/brain/awh006 14607796

[B145] Olsson C. J. (2012). Complex motor representations may not be preserved after complete spinal cord injury. Exp. Neurol. 236, 46–49 10.1016/j.expneurol.2012.03.022 22504114

[B146] Pascual-Leone A. Peris M. Tormos J. M. Pascual A. P. Catala M. D. (1996). Reorganization of human cortical motor output maps following traumatic forearm amputation. Neuroreport 7, 2068–2070 10.1097/00001756-199609020-00002 8930960

[B147] Patuzzo S. Fiaschi A. Manganotti P. (2003). Modulation of motor cortex excitability in the left hemisphere during action observation: a single- and paired-pulse transcranial magnetic stimulation study of self- and non-self-action observation. Neuropsychologia 41, 1272–1278 10.1016/S0028-3932(02)00293-2 12753966

[B148] Personnier P. Paizis C. Ballay Y. Papaxanthis C. (2008). Mentally represented motor actions in normal aging II. The influence of the gravito-inertial context on the duration of overt and covert arm movements. Behav. Brain Res. 186, 273–283 10.1016/j.bbr.2007.08.018 17913253

[B149] Porro C. A. Cettolo V. Francescato M. P. Baraldi P. (2000). Ipsilateral involvement of primary motor cortex during motor imagery. Eur. J. Neurosci. 12, 3059–3063 10.1046/j.1460-9568.2000.00182.x 10971647

[B150] Porro C. A. Francescato M. P. Cettolo V. Diamond M. E. Baraldi P. Zuiani C. Bazzocchi M. Di Prampero P. E. (1996). Primary motor and sensory cortex activation during motor performance and motor imagery: a functional magnetic resonance imaging study. J. Neurosci. 16, 7688–7698 8922425 10.1523/JNEUROSCI.16-23-07688.1996 PMC6579073

[B151] Quartarone A. Bagnato S. Rizzo V. Morgante F. Sant’Angelo A. Crupi D. Romano M. Messina C. Berardelli A. Girlanda P. (2005). Corticospinal excitability during motor imagery of a simple tonic finger movement in patients with writer’s cramp. Mov. Disord. 20, 1488–1495 10.1002/mds.20626 16078218

[B152] Raffin E. Giraux P. Reilly K. T. (2012a). The moving phantom: motor execution or motor imagery? Cortex 48, 746–757 10.1016/j.cortex.2011.02.003 21397901

[B153] Raffin E. Mattout J. Reilly K. T. Giraux P. (2012b). Disentangling motor execution from motor imagery with the phantom limb. Brain 135, 582–595 10.1093/brain/awr337 22345089

[B154] Ramachandran V. S. Brang D. Mcgeoch P. D. (2010). Dynamic reorganization of referred sensations by movements of phantom limbs. Neuroreport 21, 727–730 20523250 10.1097/WNR.0b013e32833be9ab

[B155] Ranganathan V. K. Siemionow V. Liu J. Z. Sahgal V. Yue G. H. (2004). From mental power to muscle power-gaining strength by using the mind. Neuropsychologia 42, 944–956 10.1016/j.neuropsychologia.2003.11.018 14998709

[B156] Reilly K. T. Mercier C. Schieber M. H. Sirigu A. (2006). Persistent hand motor commands in the amputees’ brain. Brain 129, 2211–2223 10.1093/brain/awl154 16799174

[B157] Reis J. Swayne O. B. Vandermeeren Y. Camus M. Dimyan M. A. Harris-Love M. Perez M. A. Ragert P. Rothwell J. C. Cohen L. G. (2008). Contribution of transcranial magnetic stimulation to the understanding of cortical mechanisms involved in motor control. J. Physiol. (Lond.) 586, 325–351 10.1113/jphysiol.2007.144824 17974592 PMC2375593

[B158] Roosink M. Zijdewind I. (2010). Corticospinal excitability during observation and imagery of simple and complex hand tasks: implications for motor rehabilitation. Behav. Brain Res. 213, 35–41 10.1016/j.bbr.2010.04.027 20433871

[B159] Ross J. S. Tkach J. Ruggieri P. M. Lieber M. Lapresto E. (2003). The mind’s eye: functional MR imaging evaluation of golf motor imagery. AJNR Am. J. Neuroradiol. 24, 1036–1044 12812924 PMC8149015

[B160] Rossini P. M. Rossi S. Pasqualetti P. Tecchio F. (1999). Corticospinal excitability modulation to hand muscles during movement imagery. Cereb. Cortex 9, 161–167 10.1093/cercor/9.2.161 10220228

[B161] Rothwell J. C. (1991). Physiological studies of electric and magnetic stimulation of the human brain. Electroencephalogr. Clin. Neurophysiol. Suppl. 43, 29–35 1773767

[B162] Roux F. E. Ibarrola D. Lazorthes Y. Berry I. (2001). Virtual movements activate primary sensorimotor areas in amputees: report of three cases. Neurosurgery 49, 736–741; discussion 741–732. 10.1097/00006123-200109000-00039 11523688

[B163] Roux F. E. Lotterie J. A. Cassol E. Lazorthes Y. Sol J. C. Berry I. (2003). Cortical areas involved in virtual movement of phantom limbs: comparison with normal subjects. Neurosurgery 53, 1342–1352; discussion 1352–1343. 14633300 10.1227/01.neu.0000093424.71086.8f

[B164] Roy F. D. Zewdie E. T. Gorassini M. A. (2011). Short-interval intracortical inhibition with incomplete spinal cord injury. Clin. Neurophysiol. 122, 1387–1395 10.1016/j.clinph.2010.11.020 21295518

[B165] Sabate M. Gonzalez B. Rodriguez M. (2004). Brain lateralization of motor imagery: motor planning asymmetry as a cause of movement lateralization. Neuropsychologia 42, 1041–1049 10.1016/j.neuropsychologia.2003.12.015 15093143

[B166] Sabate M. Gonzalez B. Rodriguez M. (2007). Adapting movement planning to motor impairments: the motor-scanning system. Neuropsychologia 45, 378–386 10.1016/j.neuropsychologia.2006.06.025 16914174

[B167] Sabbah P. De S. S. Leveque C. Gay S. Pfefer F. Nioche C. Sarrazin J. L. Barouti H. Tadie M. Cordoliani Y. S. (2002). Sensorimotor cortical activity in patients with complete spinal cord injury: a functional magnetic resonance imaging study. J. Neurotrauma 19, 53–60 10.1089/089771502753460231 11852978

[B168] Samuel M. Ceballos-Baumann A. O. Boecker H. Brooks D. J. (2001). Motor imagery in normal subjects and Parkinson’s disease patients: an H215O PET study. Neuroreport 12, 821–828 10.1097/00001756-200103260-00040 11277590

[B169] Saturno E. Bonato C. Miniussi C. Lazzaro V. Callea L. (2008). Motor cortex changes in spinal cord injury: a TMS study. Neurol. Res. 30, 1084–1085 10.1179/174313208X332968 18768107

[B170] Schwoebel J. Boronat C. B. Branch Coslett H. (2002). The man who executed “imagined” movements: evidence for dissociable components of the body schema. Brain Cogn. 50, 1–16 10.1016/S0278-2626(02)00005-2 12372347

[B171] Sharma N. Baron J. C. Rowe J. B. (2009a). Motor imagery after stroke: relating outcome to motor network connectivity. Ann. Neurol. 66, 604–616 10.1002/ana.21810 19938103 PMC3791355

[B172] Sharma N. Simmons L. H. Jones P. S. Day D. J. Carpenter T. A. Pomeroy V. M. Warburton E. A. Baron J. C. (2009b). Motor imagery after subcortical stroke: a functional magnetic resonance imaging study. Stroke 40, 1315–1324 10.1161/STROKEAHA.108.525766 19182071

[B173] Sharma N. Jones P. S. Carpenter T. A. Baron J. C. (2008). Mapping the involvement of BA 4a and 4p during motor imagery. Neuroimage 41, 92–99 10.1016/j.neuroimage.2008.02.009 18358742

[B174] Sharma N. Pomeroy V. M. Baron J. C. (2006). Motor imagery: a backdoor to the motor system after stroke? Stroke 37, 1941–1952 10.1161/01.STR.0000226902.43357.fc 16741183

[B175] Shaw W. A. (1938). The distribution of muscular action potentials during imaging. Psychol. Rec. 2, 195–216

[B176] Shick J. (1970). Effects of mental practice on selected volleyball skills for college women. Res. Q. 41, 88–94 5266498

[B177] Shukla G. D. Sahu S. C. Tripathi R. P. Gupta D. K. (1982). Phantom limb: a phenomenological study. Br. J. Psychiatry 141, 54–58 10.1192/bjp.141.1.97 7116073

[B178] Sirigu A. Cohen L. Duhamel J. R. Pillon B. Dubois B. Agid Y. Pierrot-Deseilligny C. (1995). Congruent unilateral impairments for real and imagined hand movements. Neuroreport 6, 997–1001 10.1097/00001756-199505090-00012 7632907

[B179] Sirigu A. Duhamel J. R. Cohen L. Pillon B. Dubois B. Agid Y. (1996). The mental representation of hand movements after parietal cortex damage. Science 273, 1564–1568 10.1126/science.273.5281.1564 8703221

[B180] Slade J. M. Landers D. M. Martin P. E. (2002). Muscular activity during real and imagined movements: a test of inflow explanations. J. Sport Exerc. Psychol. 24, 151–167

[B181] Solodkin A. Hlustik P. Chen E. E. Small S. L. (2004). Fine modulation in network activation during motor execution and motor imagery. Cereb. Cortex 14, 1246–1255 10.1093/cercor/bhh086 15166100

[B182] Stinear C. M. (2010). “Corticospinal facilitation during motor imagery,” in The Neurophysiological Foundations of Mental and Motor Imagery, eds Guillot A. Collet C. (New York, NY: Oxford University Press), 47–61

[B183] Stinear C. M. Byblow W. D. (2003a). Motor imagery of phasic thumb abduction temporally and spatially modulates corticospinal excitability. Clin. Neurophysiol. 114, 909–914 10.1016/S1388-2457(02)00373-5 12738438

[B184] Stinear C. M. Byblow W. D. (2003b). Role of intracortical inhibition in selective hand muscle activation. J. Neurophysiol. 89, 2014–2020 10.1152/jn.00925.2002 12611950

[B185] Stinear C. M. Byblow W. D. (2004). Modulation of corticospinal excitability and intracortical inhibition during motor imagery is task-dependent. Exp. Brain Res. 157, 351–358 10.1007/s00221-004-1851-z 14997259

[B186] Stinear C. M. Byblow W. D. Steyvers M. Levin O. Swinnen S. P. (2006). Kinesthetic, but not visual, motor imagery modulates corticomotor excitability. Exp. Brain Res. 168, 157–164 10.1007/s00221-005-0078-y 16078024

[B187] Stinear C. M. Coxon J. P. Byblow W. D. (2009). Primary motor cortex and movement prevention: where Stop meets Go. Neurosci. Biobehav. Rev. 33, 662–673 10.1016/j.neubiorev.2008.08.013 18789963

[B188] Stinear C. M. Fleming M. K. Barber P. A. Byblow W. D. (2007). Lateralization of motor imagery following stroke. Clin. Neurophysiol. 118, 1794–1801 10.1016/j.clinph.2007.05.008 17581773

[B189] Subramanian L. Hindle J. V. Johnston S. Roberts M. V. Husain M. Goebel R. Linden D. (2011). Real-time functional magnetic resonance imaging neurofeedback for treatment of Parkinson’s disease. J. Neurosci. 31, 16309–16317 10.1523/JNEUROSCI.6594-10.2011 22072682 PMC6633236

[B190] Suinn R. M. (1980). “Body thinking: psychology for Olympic champions,” in Psychology in Sports: Methods and Applications, ed. Suinn R. M. (Mineapolis: Burgess), 306–315

[B191] Szameitat A. J. Shen S. Conforto A. Sterr A. (2012). Cortical activation during executed, imagined, observed, and passive wrist movements in healthy volunteers and stroke patients. Neuroimage 62, 266–280 10.1016/j.neuroimage.2012.05.009 22584231

[B192] Tamir R. Dickstein R. Huberman M. (2007). Integration of motor imagery and physical practice in group treatment applied to subjects with Parkinson’s disease. Neurorehabil. Neural Repair 21, 68–75 10.1177/1545968306292608 17172556

[B193] Taniguchi S. Kimura J. Yamada T. Ichikawa H. Hara M. Fujisawa R. Shimizu H. Tani T. (2008). Effect of motion imagery to counter rest-induced suppression of F-wave as a measure of anterior horn cell excitability. Clin. Neurophysiol. 119, 1346–1352 10.1016/j.clinph.2007.11.179 18396453

[B194] Thobois S. Dominey P. Fraix V. Mertens P. Guenot M. Zimmer L. Pollak P. Benabid A. L. Broussolle E. (2002). Effects of subthalamic nucleus stimulation on actual and imagined movement in Parkinson’s disease: a PET study. J. Neurol. 249, 1689–1698 10.1007/s00415-002-0906-y 12529791

[B195] Thobois S. Dominey P. F. Decety J. Pollak P. P. Gregoire M. C. Le Bars P. D. Broussolle E. (2000). Motor imagery in normal subjects and in asymmetrical Parkinson’s disease: a PET study. Neurology 55, 996–1002 10.1212/WNL.55.7.996 11061258

[B196] Topka H. Cohen L. G. Cole R. A. Hallett M. (1991). Reorganization of corticospinal pathways following spinal cord injury. Neurology 41, 1276–1283 10.1212/WNL.41.8.1276 1866018

[B197] Tremblay F. Leonard G. Tremblay L. (2008). Corticomotor facilitation associated with observation and imagery of hand actions is impaired in Parkinson’s disease. Exp. Brain Res. 185, 249–257 10.1007/s00221-007-1150-6 17926025

[B198] Verbruggen F. Logan G. D. (2009). Models of response inhibition in the stop-signal and stop-change paradigms. Neurosci. Biobehav. Rev. 33, 647–661 10.1016/j.neubiorev.2008.08.014 18822313 PMC2696813

[B199] Vromen A. Verbunt J. A. Rasquin S. Wade D. T. (2011). Motor imagery in patients with a right hemisphere stroke and unilateral neglect. Brain Inj. 25, 387–393 10.3109/02699052.2011.558041 21355672

[B200] Watanabe J. Sugiura M. Sato K. Sato Y. Maeda Y. Matsue Y. Fukuda H. Kawashima R. (2002). The human prefrontal and parietal association cortices are involved in NO-GO performances: an event-related fMRI study. Neuroimage 17, 1207–1216 10.1006/nimg.2002.1198 12414261

[B201] Wehner T. Vogt S. Stadler M. (1984). Task-specific EMG-characteristics during mental training. Psychol. Res. 46, 389–401 10.1007/BF00309071 6522565

[B202] Wolf S. L. Blanton S. Baer H. Breshears J. Butler A. J. (2002). Repetitive task practice: a critical review of constraint-induced movement therapy in stroke. Neurologist 8, 325–338 10.1097/00127893-200211000-00001 12801434 PMC3572508

[B203] Wu A. J. Hermann V. Ying J. Page S. J. (2010). Chronometry of mentally versus physically practiced tasks in people with stroke. Am. J. Occup. Ther. 64, 929–934 10.5014/ajot.2010.09005 21218684 PMC3245978

[B204] Yahagi S. Kasai T. (1998). Facilitation of motor evoked potentials (MEPs) in first dorsal interosseous (FDI) muscle is dependent on different motor images. Electroencephalogr. Clin. Neurophysiol. 109, 409–417 10.1016/S0924-980X(98)00041-1 9851298

[B205] Yahagi S. Shimura K. Kasai T. (1996). An increase in cortical excitability with no change in spinal excitability during motor imagery. Percept. Mot. Skills 83, 288–290 10.2466/pms.1996.83.1.288 8873203

[B206] Yue G. Cole K. J. (1992). Strength increases from the motor program: comparison of training with maximal voluntary and imagined muscle contractions. J. Neurophysiol. 67, 1114–1123 1597701 10.1152/jn.1992.67.5.1114

[B207] Zatsiorsky V. M. Li Z. M. Latash M. L. (2000). Enslaving effects in multi-finger force production. Exp. Brain Res. 131, 187–195 10.1007/s002219900261 10766271

[B208] Zijdewind J. Toering S. T. Bessen B. Van-Der-Laan O. Diercks R. L. (2003). Effects of imagery motor training on torque production of ankle plantar flexor muscles. Muscle Nerve 28, 168–173 10.1002/mus.10406 12872320

[B209] Zimmermann-Schlatter A. Schuster C. Puhan M. A. Siekierka E. Steurer J. (2008). Efficacy of motor imagery in post-stroke rehabilitation: a systematic review. J. Neuroeng. Rehabil. 5, 8 10.1186/1743-0003-5-8 18341687 PMC2279137

